# Long-Term Outcomes and Safety of Non-Cyclodialytic, Bleb-Independent Surgical Enhancement of Uveoscleral Outflow in Open-Angle Glaucoma, with Evaluation of the Role of Conjunctival Lymphatic Vessels in Suprachoroidal Drainage

**DOI:** 10.3390/vision10040084

**Published:** 2026-10-15

**Authors:** Vinod Kumar, Mikhail Aleksandrovich Frolov, Galina Nikolaevna Dushina, Andrey Igorevich Bezzabotnov, Ahmad Saleh Soliman Shradqa, Zarina Shaykuliyevna Rustamova

**Affiliations:** 1 Department of Eye Diseases, Medical Institute, Patrice Lumumba Peoples’ Friendship University of Russia, 6 Mikluho-Maklaya St., 117198 Moscow, Russia; frolov-ma@rudn.ru (M.A.F.); dushina-gn@rudn.ru (G.N.D.); 2 Centre of Eye Microsurgery “PRO Zrenie”, 1 Gorshina Str., 141407 Khimki, Russia; magnawer@yandex.ru (A.I.B.); sh1988moscow@gmail.com (A.S.S.S.); 3 Independent Researcher, 2297 street, building 18, Sultangazi Area, 34265 Istanbul, Türkiye; shaykuliyevna0294@gmail.com

**Keywords:** open-angle glaucoma, collagen implant, glaucoma surgery, suprachoroidal drainage, uveoscleral outflow, conjunctival lymphatic vessels, SCS–scleral–episcleral–CLV route

## Abstract

**Purpose:** To evaluate long-term outcomes and safety of non-cyclodialytic, bleb-independent surgical enhancement of uveoscleral outflow for intraocular pressure (IOP) reduction in open-angle glaucoma (OAG), and to assess the role of conjunctival lymphatic vessels (CLVs) in suprachoroidal drainage. **Methods:** In this single-center consecutive case series, 70 eyes of 63 patients (mean age 75.0 years; 70% female) with moderate-to-severe OAG underwent uveoscleral outflow enhancement. Twenty-two eyes had standalone surgery; 48 eyes had combined phacoemulsification with glaucoma surgery. Mean follow-up was 24.1 months (range 5.3–60.4). Primary outcomes were IOP reduction and surgical success by Kaplan–Meier analysis (sustained failure: IOP > 18 mmHg or < 20% reduction from baseline at two consecutive available postoperative visits, excluding day 1). CLVs were assessed by slit-lamp and OCT at 11 time points over 36 months. Mixed-effects models with random intercept per eye were used for repeated measurements. Logistic regression with cluster-robust standard errors evaluated predictors of CLV visibility. **Results:** Baseline IOP of 28.8 ± 8.4 mmHg decreased to 12.3 ± 4.5 mmHg at 1 month (54.5% reduction) and 14.5 ± 2.5 mmHg at 36 months (46.2% reduction, n = 22). Medications decreased from 2.77 ± 0.94 to 1.36 ± 1.09 at 36 months. Overall surgical survival was 91.4% at 1 month, 82.0% at 12 months, and 74.1% at 36 months. Fifteen eyes (21.4%) met sustained failure criteria (standalone surgery group: eight, combined surgery group: seven; and log-rank *p* = 0.038). The combined surgery group showed higher survival at 36 months (82.6% vs. 58.5%), but the difference did not reach significance. CLVs were detected in 51.4% by slit-lamp and 96.4% by OCT, peaking at 1 month. Before surgery, CLVs were not detected on OCT in any eye. Time was a significant negative predictor of CLV visibility on both slit-lamp (OR 0.953/month, *p* = 0.046) and OCT (OR 0.935/month, *p* = 0.003), without independent association with IOP or medication burden, suggesting that CLVs facilitate suprachoroidal drainage as a structural mechanism rather than a reactive response to IOP changes. **Conclusions:** Non-cyclodialytic, bleb-independent uveoscleral outflow enhancement provides sustained IOP reduction with favorable safety. CLVs, nearly universally detected by OCT after surgery, appear to contribute to suprachoroidal drainage. Combined surgery showed a trend toward greater surgical success than standalone procedures.

## 1. Introduction

In the treatment of open-angle glaucoma (OAG), intraocular pressure (IOP) remains the sole therapeutic target of available IOP-lowering agents, laser modalities, and surgical techniques. Physiologically, aqueous humor (AH) exits the anterior chamber (AC) through the following two major drainage routes: the conventional outflow pathway (via the trabecular meshwork) and the non-conventional, non-trabecular pathways [1].

Although the non-trabecular outflow pathway holds great potential for lowering IOP in glaucoma patients, it remains one of the least studied areas to date. Historically, cyclodialysis—the detachment of the ciliary body from the scleral spur to create a cyclodialysis cleft—was the main surgical approach for glaucoma before trabeculectomy was introduced (Heine L., 1905) [2]. While this procedure effectively reduced IOP, its long-term outcomes were inconsistent, largely because the cleft tended to close over time. Several implants and scaffolds have been developed to keep the cleft open, but their success has been limited [3,4,5,6,7]. Detaching the ciliary body is inherently traumatic and often triggers inflammation and fibrosis at the surgical site, which likely explains why the cleft closes. These limitations highlight the need for new surgical techniques that can redirect AH from the AC to the suprachoroidal space (SCS) without performing cyclodialysis, while also preventing AH leakage into the subconjunctival space.

To achieve these objectives, a two-stage surgical approach was developed [8]. In the first stage, an intrascleral chamber (ISC) was created by excising a block of deep scleral tissue together with the external wall of Schlemm’s canal (SC). No window was made in Descemet’s membrane, thereby avoiding filtration during the early postoperative period. A biodegradable collagen implant (BCI) was then positioned with one end in the ISC and the other in the SCS, serving as both a spacer and a conduit. In the second stage, Nd:YAG laser trabeculotomy (LT) was performed postoperatively to allow AH to flow from the AC into the intrascleral pool and subsequently into the SCS. This technique proved highly effective in lowering IOP without forming a filtration bleb (FB), as confirmed by optical coherence tomography (OCT). During early postoperative follow-ups, an unexpected finding was noted: on slit-lamp examination, transparent conjunctival lymphatic vessels (CLVs) of various forms and shapes became visible in the superior bulbar conjunctiva in approximately 50% of cases. The efficacy and safety of this technique were subsequently evaluated in a consecutive case series of 38 patients, with comparable results [9]. These observations led to a hypothesis: cyclodialysis-independent enhancement of the non-trabecular outflow pathway activates not only the uveoscleral, uveovortex, and uveolymphatic routes but also a novel drainage pathway—the SCS–scleral–episcleral–CLV route. The present interventional consecutive case series reports the long-term clinical outcomes of this technique to further support this hypothesis.

## 2. Materials and Methods

This was a single-center, interventional, consecutive case series without a control arm. Data were obtained through a retrospective review of medical records. We reviewed the medical charts of patients from whom informed consent had been obtained and had open-angle glaucoma (OAG). There are records of 98 patients (105 eyes) who underwent a surgical procedure to enhance uveoscleral outflow by rerouting AH from the AC to the SCS without detaching the ciliary body from the scleral spur. The procedures were performed between 1 March 2020 and 31 March 2025. Of these, 63 patients (70 eyes) met the predefined inclusion criteria and were included in the analysis. In 22 eyes (31.4%), the procedure was performed as a standalone intervention; in 48 eyes (68.6%), it was combined with cataract surgery. All surgeries were performed by a single surgeon (VK).

Eligible eyes were either phakic (with or without a visually significant cataract) or pseudophakic. Patients were eligible if they met at least one of the following criteria: medically uncontrolled IOP, non-compliance with prescribed IOP-lowering medication, or decompensated IOP after prior glaucoma surgery. Each patient was required to have at least 6 months of follow-up; longitudinal data were collected for up to 36 months post-baseline.

**Figure 1:**
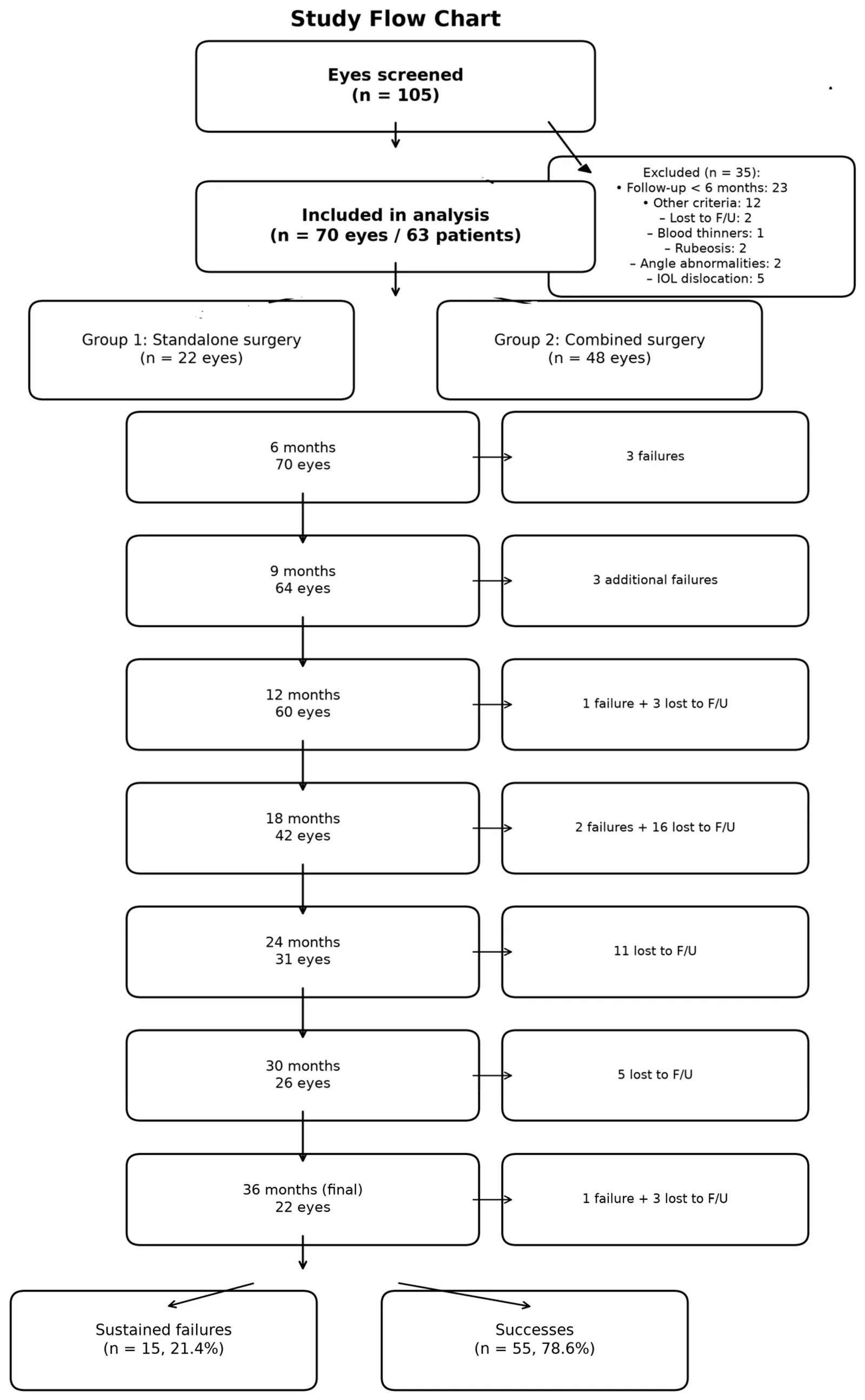
Flow chart of patient screening, inclusion and exclusion criteria, and surgical outcomes.

Patients were excluded if their medical charts revealed any of the following: diagnosis of narrow-angle, closed-angle, neovascular, congenital, phacolytic, or phacomorphic glaucoma; an acute glaucoma attack; intraocular lens (IOL) dislocation; ocular pathologies such as rubeosis or angle abnormalities; any significant ocular comorbidity or ocular pathology that could compromise the accuracy of IOP measurement; inability to discontinue blood-thinning agents; or a history of uveitis or ocular infection in either eye within 90 days prior to screening. Details of screening, inclusion, and exclusion of patients and eyes are shown in flow chart (Figure 1).

Each patient received a full preoperative ophthalmic workup. Visual acuity (VA) was measured with a Snellen chart and converted to logMAR for statistical analysis. Other assessments included tonometry, perimetry, and ophthalmoscopy. Spectral-domain OCT was performed using a SOCT Copernicus Revo 80 (OPTOPOL Technology Sp.z o.o., 42-400 Zawiercie, Poland). When the native lens condition allowed sufficient image quality, detailed glaucoma analysis was carried out, covering RNFL thickness, ONH morphology, DDLS, and ganglion cell layers (RNFL + GCL + IPL and GCL + IPL), in both eyes and including hemisphere asymmetry. In addition, OCT of the superior bulbar conjunctiva was obtained in every patient to detect CLVs.

Patients continued their preoperative hypotensive regimens unchanged. Prophylactic eye drops were started 3 days before surgery: levofloxacin 0.5% (1–2 drops t.i.d.) and bromfenac 0.09% (once daily). For analysis, glaucoma medications were counted as separate agents, with combination drops counted as two if they contained two active compounds.

The study protocol was approved by the research Ethics Committee of the Medical Institute of the Patrice Lumumba People’s Friendship University of Russia, Moscow, and complied with the principles of the Declaration of Helsinki. Written informed consent was obtained from all patients included in the study.

### 2.1. Surgical Technique

**Figure 2:**
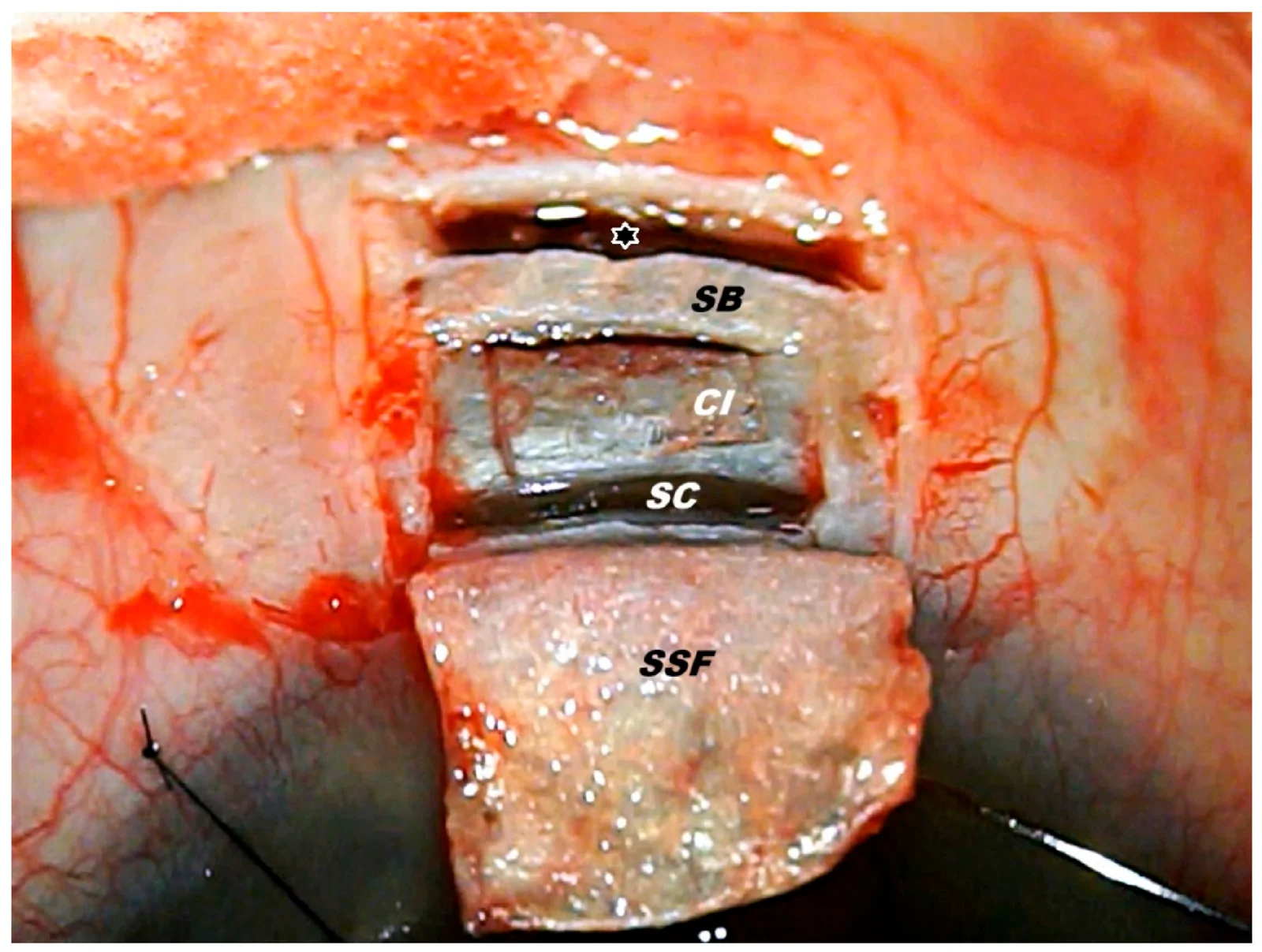
This figure illustrates a case of combined surgery. After phacoemulsification and intraocular lens implantation, the phaco incision was closed with an interrupted suture. The figure depicts key intraoperative landmarks: the superficial scleral flap (SSF) and the exposed inner wall of SC. A transparent biodegradable CI rests on the residual sclera above the uvea. Its anterior end is positioned 1 mm from the inner wall of Schlemm’s canal, and its posterior end extends into the suprachoroidal tunnel. Complete excision of the proximal deep scleral segment has exposed a broader uveal surface for AH drainage (indicated by a white star with black borders). The CI is stabilized by the middle portion of the “scleral bridge” (SB) without requiring any suture fixation.

The surgical technique has been described in detail elsewhere [8,9]. Retrobulbar anesthesia was used. A traction suture was placed at the limbus at either the 12 o’clock or 6 o’clock position. In standalone cases, before performing the glaucoma procedure, a 1.5–2.0 mm clear corneal incision was made, and the AC was partially filled with a cohesive viscoelastic device (1.0–1.4% hyaluronic acid solution). Peripheral iridotomy was performed using a capsulorhexis needle with the Oertli CataRhex 3 apparatus (Supplementary Video S1). The viscoelastic device was then irrigated out and the incision hydrated. After a fornix-based conjunctival peritomy, a 4 × 4 mm, approximately one-third-thickness, limbal-based superficial scleral flap was fashioned. Parallel to the limbus at 2, 3, and 4 mm posterior to it, three transverse incisions were made through the deep scleral layers down to the uveal tissue, dividing the deep sclera into three segments. In the distal segment, deep scleral tissue was excised together with the external wall of SC, leaving a thin layer of sclera over the ciliary body. No window was created in Descemet’s membrane. Part of the juxtacanalicular connective tissue was stripped from the inner wall of the SC and removed. Beneath the middle and proximal scleral segments, the uvea was gently detached from the overlying sclera using a thin, blunt spatula. A strip of BCI (MakMedi glaucoma drainage, Russia) was then inserted into the cleft with forceps, with its anterior end positioned in the ISC to serve as a spacer and conduit. The proximal scleral segment was completely excised, exposing a wider uveal surface area for contact with AH (Figure 2). Finally, the superficial scleral flap was repositioned and closed in a watertight fashion with five interrupted 10-0 nylon sutures, and the conjunctiva was sutured back to the limbus (Supplementary Video S2).

A two-site approach was used for combined surgery. First, cataract surgery was performed. After IOL implantation, the viscoelastic device was irrigated out, and pupillary constriction was achieved by irrigating the AC with 0.01% carbachol solution (sol. Miocol 0.01%, Appasamy Ocular Devices, Chennai, Tamil Nadu, India). The AC was then partially refilled with viscoelastic, and a peripheral iridotomy was created using a capsulorhexis needle with the CataRhex 3 apparatus (Oertli Instruments, Hori, Switzerland). The viscoelastic was subsequently removed from the AC via irrigation and aspiration, and the main incision was closed with an interrupted suture. Glaucoma surgery was then performed at the 12 o’clock position. In patients with a history of prior glaucoma procedures, the intervention was carried out in areas adjacent to the previous surgical site to avoid disturbing it.

**Figure 3:**
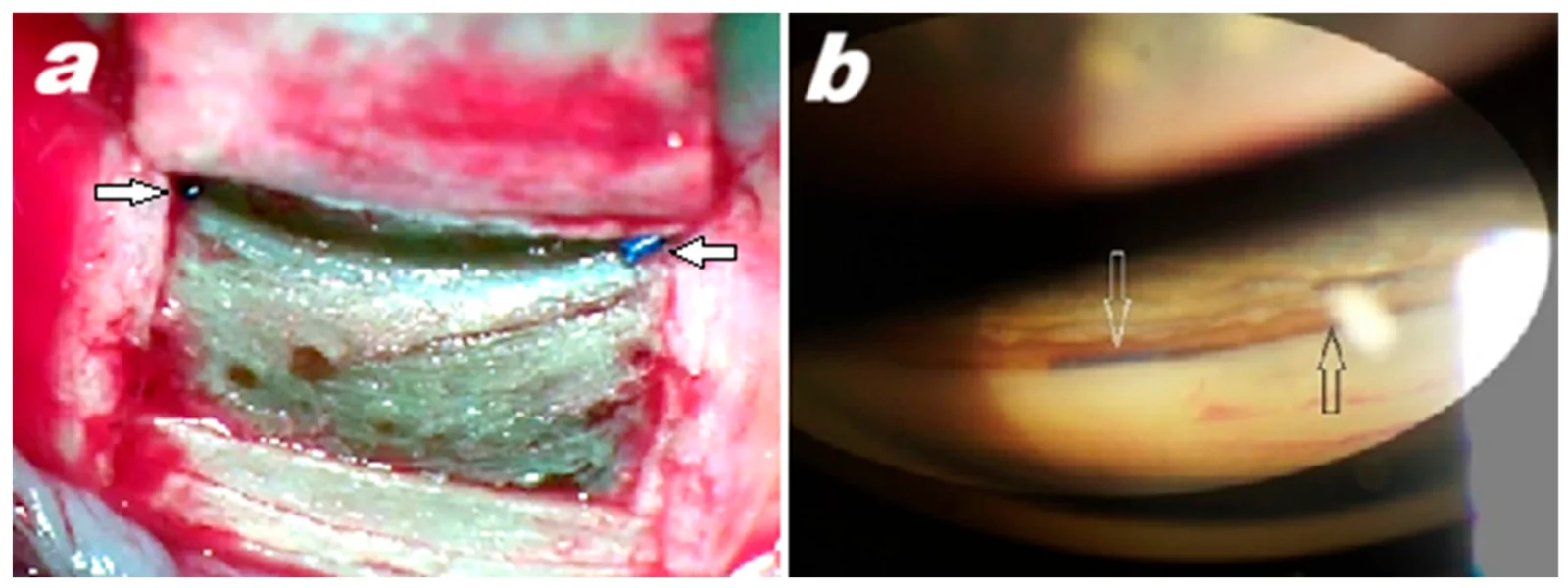
Insertion of polypropylene suture markers in SC to localize the surgical site. (**a**) Intraoperative view showing two small pieces (0.5 cm each) of 4-0 polypropylene suture inserted into both open ends of SC (white arrows with black borders). (**b**) Gonioscopic view of the AC angle demonstrating the polypropylene suture pieces (white arrows with black borders) within the lumen of SC. The segment of SC between the two markers corresponds to the surgical site, where LT was performed safely and effectively using minimal energy.

Postoperatively, in some cases, the exact location of the surgical site was difficult to identify on gonioscopy. To address this, the technique was modified: two small pieces of 4-0 or 5-0 polypropylene suture (0.5 cm each) were inserted into both open ends of SC (Supplementary Video S3). This maneuver facilitated identification of the surgical site during gonioscopy (Figure 3a,b). Additionally, the “scleral bridge” (SB) was further thinned, leaving approximately 25% of the original scleral thickness over the uveal tissue. This modification increased the contact surface area between the uvea and the AH.

Primary outcome was proportion of eyes with final IOP < 18 mm Hg and ≥ 20% IOP reduction from baseline, maintained on the same or fewer medications.

Secondary outcomes: mean IOP change at each follow-up and change in number of IOP-lowering medications versus baseline.

The safety profile was assessed by recording any intraoperative or postoperative complications, the need for further glaucoma surgery during follow-up, and any reduction in best-corrected VA of two or more lines compared with baseline values.

All patients followed a standardized postoperative protocol. Topical dexamethasone 0.1% was prescribed with a tapering schedule: 3 times daily for the first week, reduced to twice daily in the second week, and once daily in the third week. Antibiotic coverage consisted of either moxifloxacin 0.5% or levofloxacin 0.5% solution, given 3 times daily for 7 days.

A follow-up visit was scheduled for the day after surgery, during which the decision to continue or discontinue hypotensive medications was made according to the patient’s IOP. Conjunctival sutures were removed between the 7th and 10th postoperative day.

Postoperative assessment included VA, tonometry, biomicroscopy, ophthalmoscopy, gonioscopy, and OCT evaluation of the surgical site and adjacent areas. Where possible, findings were documented photographically and on video.

Follow-up visits were scheduled at the following time points: postoperative day 1, 1 week, 1, 3, 6, 9, 12, 18, 24, 30, and 36 months postoperatively. Patients with suboptimal IOP control were evaluated more frequently as clinically indicated.

In cases of elevated IOP, LT was performed using a slit-lamp-mounted Nd:YAG laser (Optotek Medical optoYAG, Zgornji Brnik, Slovenia) and a single-mirror laser goniolens (Ocular Latina Gonio Laser Lens, Ocular Instruments, Bellevue, WA, USA). The procedure was undertaken no earlier than 7–10 days after surgery, allowing the conjunctiva to heal completely and the underlying scleral tissue to prevent leakage of AH from the intrascleral chamber (ISC) into the subconjunctival space. Typically, 3–5 mJ of energy per pulse was used, and one or more openings were created in the trabecular meshwork. Successful trabeculotomy was confirmed by observing pulsatile flow of AH through the created opening. After LT, patients were prescribed bromfenac 0.09% ophthalmic solution once daily for 7–10 days.

For analysis purposes, if LT was performed within the first postoperative month, the first postoperative day was defined as the day following the laser procedure. Patients with IOP > 18 mm Hg despite LT were started on antiglaucoma medications. Those with uncontrolled IOP despite LT and maximal medical therapy were considered for additional glaucoma surgery.

CLVs were assessed at each follow-up visit using slit-lamp examination. CLV visibility was recorded as a binary outcome (visible/not visible) at 11 postoperative time points from day 1 through 36 months. Wherever possible, CLVs were photographed and video-recorded.

During OCT imaging, patients were instructed to look downward, and the upper eyelid was retracted to expose the surgical site. Five horizontal and five vertical raster scans were acquired at 1 mm intervals. Scans were assessed qualitatively. A scan was considered to be of good quality if the following structures were identifiable: the scleral flap, collagen implant, scleral lake, internal fluid-filled cavities, and AH drainage routes. Fluid-filled bleb spaces (FBs) were defined as internal fluid-filled cavities characterized by significantly low-reflective, fluid-filled spaces adjacent to the scleral flap. The hyporeflective area within the bleb was bounded by the hyperreflective conjunctiva and Tenon’s capsule. A microcyst was defined as a small, round, and hyporeflective space measuring more than 10 μm in diameter, located in the epithelial layer of the bleb wall [10].

On OCT, the CLVs were identified as hyporeflective spaces containing bicuspid valve-like structures within their lumina.

Surgical outcomes were assessed at each follow-up visit according to the following definitions:

Complete success was defined as an IOP ≤ 18 mm Hg and ≥ 20% reduction from baseline IOP; IOP-lowering medications equal to or fewer than the preoperative regimen; and loss of ≤ 2 lines of VA (LogMAR) and no additional glaucoma surgery.

Partial success was defined as an IOP ≤ 18 mm Hg and ≥20% reduction from baseline IOP; IOP-lowering medications greater than the preoperative regimen; and loss of ≤2 lines of VA (LogMAR); no additional glaucoma surgery.

Failure at a single visit was defined as any of the following: an IOP > 18 mm Hg or < 20% reduction from baseline IOP; IOP ≤ 6 mm Hg on two consecutive visits; and loss of > 2 lines of VA (LogMAR) and any additional glaucoma surgery beyond LT or iridotomy. Failure was declared only when failure criteria were met on two consecutive follow-up visits. Once an eye met failure criteria on two consecutive visits, it was classified as a permanent failure for the remainder of the study, regardless of subsequent IOP measurements or medication changes. The date of failure was recorded as the date of the second consecutive failing visit. Eyes that met failure criteria at a single visit but recovered (met complete or partial success criteria) at the next visit without additional intervention were not classified as failure. Such isolated failing visits were considered transient fluctuations and did not constitute a failure event.

Eyes were censored at the last available follow-up visit if they did not experience sustained failure during the study. Reasons for dropout—including death, loss to follow-up, and withdrawal—were documented at each time point. Eyes declared as sustained failure were not censored; they were recorded as having experienced the failure event at the date of the second consecutive failing visit.

The ophthalmic collagen implants (MakMedi, Moscow, Russia) used in this study are made from a natural biopolymer derived from porcine scleral connective tissue. They are certified medical devices, registered with the competent authorities of the country in which the study was conducted, and approved for use in glaucoma surgery in humans (Registration No. RU No. FC 01032006/3759-06, dated 23 October 2006). The implants are commercially available in various shapes and sizes; for this study, rectangular implants measuring 0.1 × 2.0 × 5.0–6.0 mm were selected. They possess a unique layered-cell structure with large interlayer spaces. When immersed in fluid, the implants swell and increase in thickness while their anterior–posterior dimensions remain essentially unchanged. According to the manufacturer, once implanted, the material is gradually resorbed by tissue fluids over time.

### 2.2. Statistics Analysis

Continuous variables are presented as mean ± SD or median (IQR), and categorical variables as frequencies and percentages. Between-group comparisons used the Mann–Whitney U test (continuous) and chi-square or Fisher’s exact test (categorical).

Surgical success was assessed at each follow-up visit using complete and qualified success criteria. Time-to-sustained-failure was analyzed by the Kaplan–Meier method, with survival curves compared using the log-rank test. Eyes without sustained failure were censored at their last visit.

To account for the repeated-measures structure and unbalanced follow-up, linear mixed-effects models were fitted for postoperative IOP and medication count. Each model included fixed effects for time (months), surgical group, and their interaction, with random intercepts and random slopes for time at the eye level. Models were estimated by maximum likelihood. The IOP model was fitted with and without adjustment for baseline IOP and age; the medication model with and without adjustment for baseline medication count. An additional IOP model included slit-lamp CLV visibility as a time-varying covariate. Fixed effects were assessed by the Wald z-test.

A generalized linear mixed model (GLMM) with a binomial distribution and logit link function was used to analyze the probability of CLV visibility on slit-lamp examination over the follow-up period. The binary outcome was CLV visibility (visible vs. not visible) assessed at each study visit. Fixed-effect predictors included time (in months), surgical group (combined surgery group vs. standalone surgery group), LT status (no vs. yes), glaucoma severity (severe vs. moderate), and patient age (centered at the mean of 75.0 years). A random intercept per eye was included to account for the correlated repeated measurements within each eye. The model was estimated using the Laplace approximation to the marginal likelihood, and standard errors were derived from the inverse of the Fisher information matrix. Odds ratios (ORs) with 95% confidence intervals (CIs) and Wald-type *p*-values are reported. The intraclass correlation coefficient (ICC) was calculated on the latent logistic scale as σ^2^/(σ^2^ + π^2^/3), where σ^2^ is the random intercept variance.

Linear mixed-effects models (LMMs) with a random intercept per eye were used to assess the association between identification of CLVs on OCT and (a) IOP and (b) medication count, adjusting for time (months), age, surgical group, glaucoma severity, and LT status. For the binary outcome (IOP > 18 mmHg), logistic regression with cluster-robust standard errors (sandwich estimator, clustered by eye) was employed as an approximation to a GLMM. A lagged analysis was performed to assess temporal directionality: OCT LV status at visit t was used to predict IOP and medications at the subsequent visit (t + 1). An OCT LV × time interaction term was included to evaluate whether the association changed over time.

Sensitivity analyses included: unadjusted models, one eye per patient, first 12 months only, beyond 12 months, early post-op (≤1 month), excluding IOP > 35 mmHg, stratification by LT status, and the lagged model. The ICC was reported to quantify between-eye variability. All analyses were performed using custom maximum likelihood estimation in Python (version 1.24, scipy.optimize).

All analyses were performed in SPSS (version 22.0.0.0; IBM Corp., Armonk, NY, USA) and independently verified using Python (NumPy version 1.24, SciPy version 1.10; Python Software Foundation). A two-tailed *p* < 0.05 was considered significant.

## 3. Results

### 3.1. Baseline Characteristics

The study included 70 eyes of 63 patients, with a mean age of 75.0 ± 7.3 years and a female predominance of 70.0%. The majority of eyes had non-refractory OAG, 75.7%, and 70.0% were classified as severe. The median follow-up duration was 17.5 months (IQR 14.1–36.6), with no significant difference between the two surgery groups (*p* = 0.761).

Patients were stratified into the following two surgery groups: standalone surgery group (n = 22) and combined surgery group (n = 48). The two groups were comparable in terms of age (76.9 ± 6.2 vs. 74.2 ± 7.6 years, *p* = 0.117), sex (68.2% vs. 70.8% female, *p* = 1.000), and laterality (45.5% vs. 47.9% right eyes, *p* = 1.000). Glaucoma severity was also similarly distributed, with severe disease present in 72.7% and 68.8% of eyes, respectively (*p* = 0.787). Glaucoma type between groups differed significantly (*p* < 0.001): combined surgery group had almost exclusively non-refractory OAG (89.6%), whereas standalone group had a substantially higher proportion of refractory OAG (54.5%). Prior glaucoma surgery was more than three times more frequent in standalone surgery group (50.0% vs. 14.6%, *p* = 0.003). Pseudophakia was nearly universal in standalone surgery group (95.5%) and entirely absent in combined surgery group (*p* < 0.001), reflecting the fundamental anatomical difference between the surgical approaches (Table 1). VA (median logMAR 0.6, IQR 0.2–1.9) and IOP (median 28.0 mmHg, IQR 24.0–31.0) were similar between the two groups (all *p* > 0.05). Demographic and baseline clinical characteristics of the study population are shown in Table 1.

### 3.2. Efficacy

#### 3.2.1. Intraocular Pressure Outcomes

Baseline IOP was comparable between the two surgery groups (standalone surgery group: 29.0 mmHg, IQR 27.0–33.8; combined surgery group: 26.5 mmHg, IQR 23.0–30.0; and *p* = 0.068), though a trend toward higher baseline pressure in standalone surgery group was observed.

**Figure 4:**
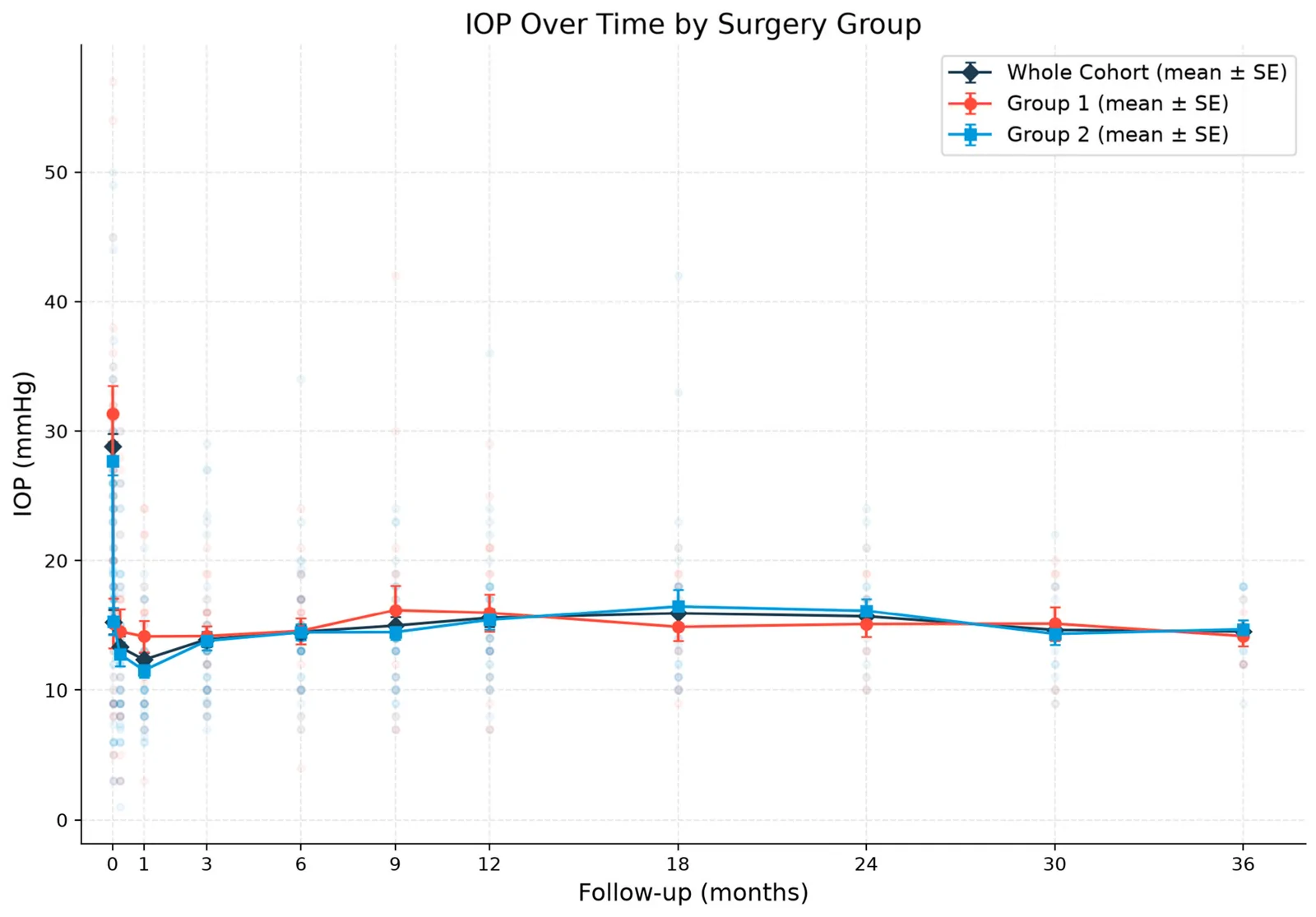
It shows IOP reduction over time in the whole cohort and by surgery groups. Group 1 = standalone surgery group, Group 2 = combined surgery group.

Following surgery, IOP decreased markedly in both groups. In the overall cohort, mean IOP fell from 28.0 mmHg at baseline to approximately 13–15 mmHg within the first postoperative day, representing a reduction of roughly 50%. This acute IOP-lowering effect was sustained throughout the follow-up period, with mean IOP remaining in the low-to-mid teens at all subsequent time points (Figure 4).

To evaluate the long-term IOP trajectory, a linear mixed-effects model with random intercept per eye was fitted, adjusting for baseline IOP. For the whole cohort, IOP showed a statistically significant decline over time, with an estimated slope of −0.068 mmHg/month (95% CI: −0.125 to −0.011, *p* = 0.020), corresponding to approximately 0.8 mmHg per year of additional IOP reduction beyond the initial postoperative drop. Baseline IOP was a significant covariate in this model (estimate 0.188, 95% CI: 0.098–0.278, and *p* < 0.001), confirming that eyes with higher starting pressure maintained higher follow-up IOP values throughout the study (Table 2).

When the two surgery groups were analyzed separately, standalone surgery group demonstrated a significant continued IOP reduction of −0.109 mmHg/month (95% CI: −0.195 to −0.023, *p* = 0.015), or approximately 1.3 mmHg per year. In combined surgery group, the IOP slope was shallower at −0.042 mmHg/month (95% CI: −0.121 to 0.036, *p* = 0.301), indicating that IOP remained essentially stable after the initial surgical effect. An interaction model was then fitted to test whether the rate of IOP change differed between groups. The time × group interaction was not statistically significant (estimate 0.076, 95% CI: −0.043 to 0.195, and *p* = 0.214), suggesting that while the direction of effect favored standalone surgery group, the difference in IOP trajectories between the two surgical approaches did not reach statistical significance. The group main effect was also non-significant (*p* = 0.317), indicating no consistent difference in absolute IOP level between groups during follow-up after adjusting for baseline pressure.

**Figure 5:**
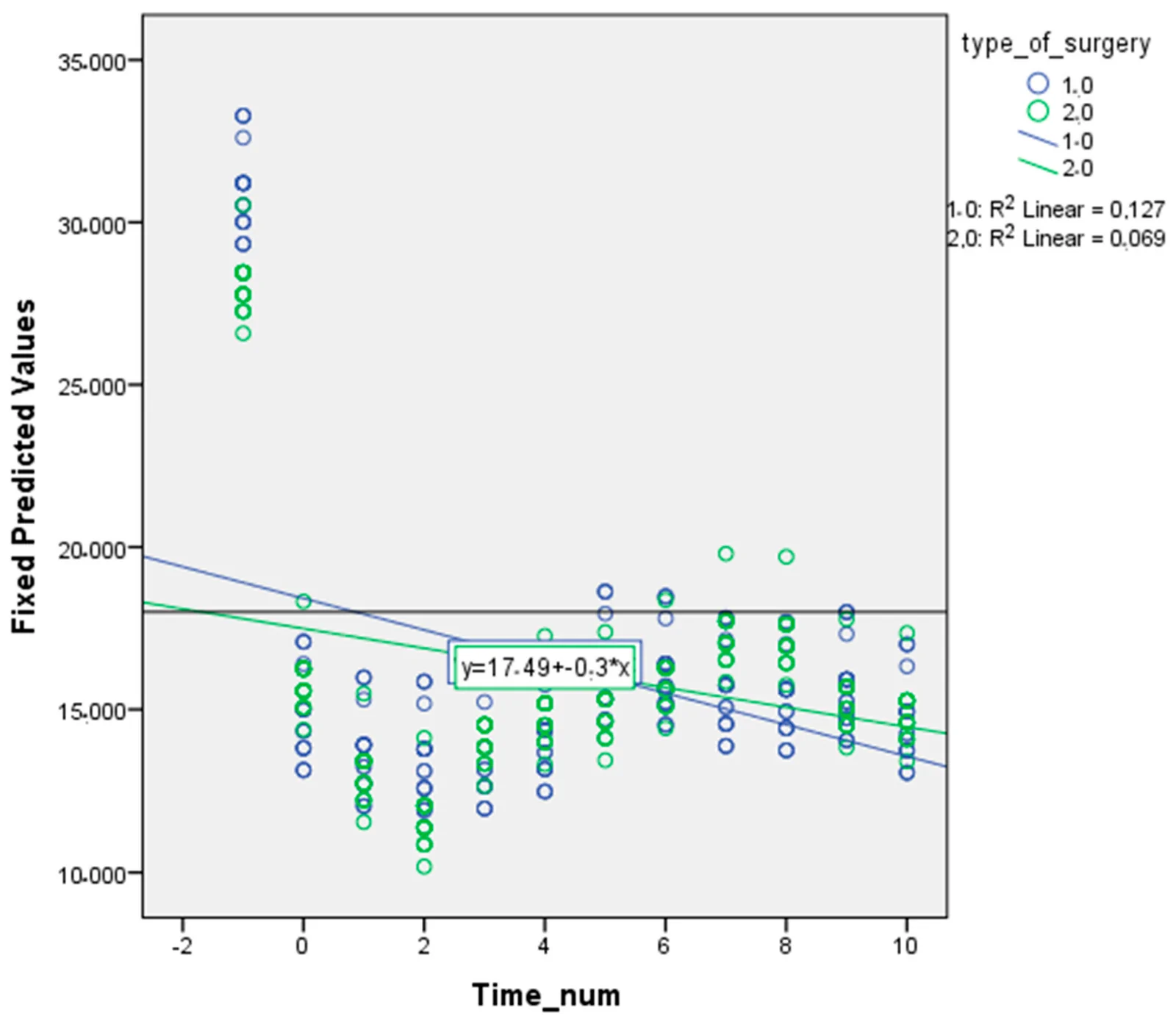
It shows the scatter plot of IOP measurements over the 12 time points (P0–P11), stratified by type of surgery. Each point represents a single eye measurement; fitted regression lines illustrate the IOP trajectory for each surgery group (blue color for standalone glaucoma surgery group; green color line—combined glaucoma and cataract surgeries group). At baseline (P0), mean IOP for all eyes was 28.8 ± 8.4 mmHg, 29 [27.3–33.8] mmHg in the standalone glaucoma surgery group and 27.7 ± 7.4 mmHg in the combined surgery group. Over the follow-up period, IOP decreased in both groups. The mixed-effects model showed a significant main effect of time (F[11, 586.835] = 47.467, *p* < 0.001), suggesting that IOP changed significantly across the follow-up period when averaged across surgery types. Blue depicted standalone glaucoma surgery and blue color depicted combined glaucoma and cataract surgery. The black line indicates 18 mmHg.

The scatter plot shows the dramatic IOP drop from baseline (~29 mmHg) to postoperative levels (~13–15 mmHg) within the first month, with IOP remaining relatively stable thereafter. Mean IOP at each time point (markers ± SE) and model-predicted trend lines are shown for both groups (Figure 5).

**Figure 6:**
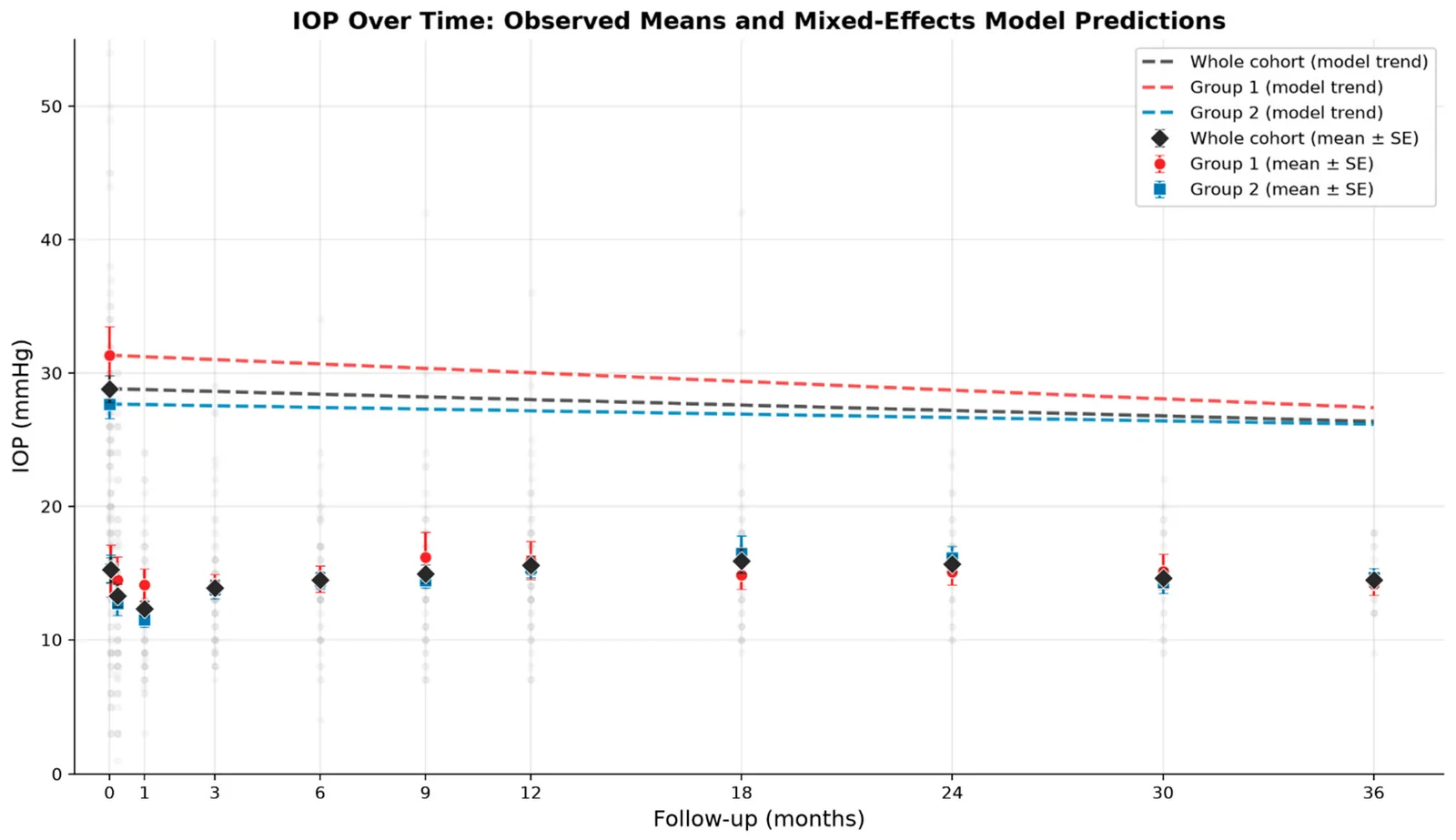
Intraocular pressure trajectories after surgery. Mean IOP (± SE) is shown at each follow-up time point for the whole cohort (black diamonds), surgery standalone surgery group (red circles), and surgery combined surgery group (blue squares). Dashed lines represent model-predicted trends from the linear mixed-effects analysis adjusted for baseline IOP. Faint gray dots indicate individual eye measurements. The figure illustrates a marked IOP reduction from baseline (approximately 28–29 mmHg) to postoperative levels (approximately 13–15 mmHg) occurring within the first day after surgery, followed by a relatively stable IOP course over the remaining follow-up period. The model-predicted trend lines indicate a slight continued decline in IOP over time for the whole cohort (−0.068 mmHg/month, *p* = 0.020) and for standalone surgery group (−0.109 mmHg/month, *p* = 0.015), while IOP in combined surgery group remained essentially stable (−0.042 mmHg/month, *p* = 0.301). The difference in slopes between the two surgery groups was not statistically significant (time × group interaction, *p* = 0.214). Group 1 = standalone surgery group, Group 2 = combined surgery group.

IOP trajectories after surgery are shown in Figure 6.

Both surgical approaches achieved a substantial and sustained IOP reduction from baseline. A modest but statistically significant continued decline in IOP was observed in the overall cohort and in standalone surgery group, while IOP in combined surgery group remained stable after the initial postoperative decrease. The difference in long-term IOP trajectories between the two groups did not reach statistical significance, though the study may have been underpowered to detect such a difference given the sample sizes.

#### 3.2.2. Medication Count Outcomes

At baseline, all eyes were on glaucoma medications, with a mean of 2.77 ± 0.94 medications and a median of 3 (IQR 2.0–3.0). The two surgery groups were comparable in terms of baseline medication burden (standalone surgery group: median 3.0, IQR 2.25–4.0; combined surgery group: median 3.0, IQR 2.0–3.0; and *p* = 0.128) (Table 1).

Following surgery, the number of glaucoma medications decreased substantially. By the first postoperative day, the mean medication count dropped to 1.04 ± 1.30, and 54.3% of eyes were medication-free. This reduction continued over the first month, reaching a mean of 0.41 ± 0.88 with 78.6% of eyes requiring no medications (*p* < 0.001 vs. baseline, paired *t*-test). The proportion of medication-free eyes peaked at 3 months (82.6%), after which a gradual increase in medication use was observed. By 12 months, 56.7% of eyes remained medication-free (mean 0.87 ± 1.16), and by 36 months, only 27.3% were medication-free (mean 1.36 ± 1.09).

**Figure 7:**
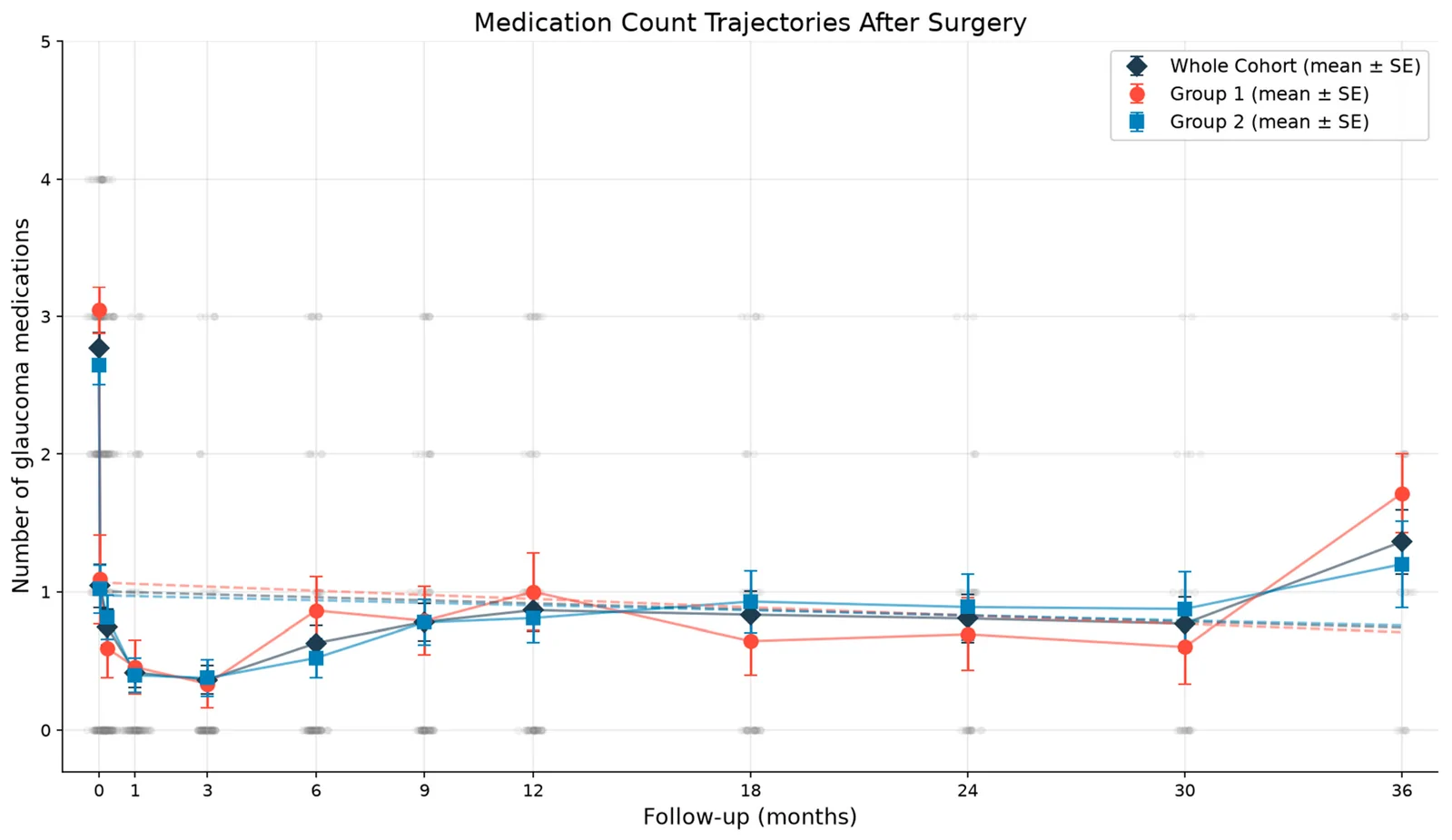
Medication count trajectories after surgery. Mean number of glaucoma medications (± SE) is shown at each follow-up time point for the whole cohort (black diamonds), standalone surgery group (red circles), and combined surgery group (blue squares). Dashed lines represent linear trend estimates. Faint gray dots indicate individual eye measurements. The figure illustrates a marked reduction in medication count from baseline (mean 2.8 medications) to near-zero levels by the first postoperative month, with 78.6% of eyes requiring no medications at that time point. Thereafter, a gradual increase in medication use is observed, with mean medication count rising to approximately 0.9 at 12 months and 1.4 at 36 months. The overall time effect was not statistically significant in the mixed-effects model (*p* = 0.273), and no significant difference was observed between the two surgery groups in either medication level (*p* = 0.858) or rate of change over time (*p* = 0.774).

A linear mixed-effects model with random intercept per eye, adjusted for baseline medication count, was used to evaluate the medication count trajectory over the full follow-up period. In the whole cohort, the time effect was not statistically significant (estimate −0.008 per month, 95% CI: −0.020 to 0.005, and *p* = 0.228), indicating that when the immediate postoperative reduction was accounted for, there was no significant linear trend in medication count over time. Baseline medication count was not a significant covariate (estimate 0.118, 95% CI: −0.021 to 0.256, and *p* = 0.096) (Figure 7).

When analyzed by surgery group, neither standalone surgery group (time estimate −0.009 per month, *p* = 0.338) nor combined surgery group (time estimate −0.007 per month, *p* = 0.447) showed a statistically significant change in medication count over time. The interaction model confirmed no significant difference between groups in either medication level (group effect *p* = 0.806) or rate of change (time × group interaction *p* = 0.828).

The graph (Figure 7) demonstrates the following: (1) a sharp decrease following surgery—from approximately 2.8 medications to near zero within one month; (2) a medication-free period lasting approximately 3 months; and (3) a gradual increase as medications are reintroduced to maintain IOP control. The trend lines are nearly flat for all groups, consistent with the non-significant mixed-effects model results—the initial decrease and the gradual increase approximately offset each other over the full follow-up period.

The results demonstrate that while both surgical approaches achieved substantial initial reductions in medication burden, there was a gradual trend toward increased medication use over time. However, a significant proportion of eyes maintained medication-free status even at long-term follow-up, with standalone surgery group showing slightly better medication control throughout the study period.

These findings suggest that while complete medication cessation may not be sustainable for all patients long-term, both surgical techniques provide meaningful reductions in medication burden for a significant proportion of patients.

### 3.3. Visual Acuity Outcomes

At baseline, the median VA (logMAR) was 0.6 (IQR 0.2–1.9) for the whole cohort and was comparable between the two surgery groups (standalone surgery group: median 0.7, IQR 0.2–1.8; combined surgery group: median 0.5, IQR 0.2–1.8; and *p* = 0.342).

A linear mixed-effects model with random intercept per eye was used to assess changes in VA over time, adjusted for baseline VA. In the whole cohort, VA remained stable throughout the follow-up period, with a time slope estimate close to zero (estimate 0.000 per month, 95% CI: −0.002 to 0.002, and *p* = 0.848). Baseline VA was a significant covariate (estimate 0.762, 95% CI: 0.612–0.912, and *p* < 0.001), indicating that eyes with worse preoperative vision maintained lower VA values throughout follow-up.

When analyzed by surgery group, neither standalone surgery group (time estimate 0.001 per month, *p* = 0.787) nor combined surgery group (time estimate −0.001 per month, *p* = 0.831) demonstrated a significant change in VA over time. The interaction model revealed no significant differences between groups in either absolute VA level (group effect *p* = 0.294) or rate of change (time × group interaction *p* = 0.442) (Table 3).

These findings demonstrate that both surgical techniques preserved preoperative VA without evidence of long-term deterioration or significant differences between the two surgical approaches.

### 3.4. Surgical Success Outcomes

#### 3.4.1. Overall Surgical Survival (Complete and Partial Success)

Kaplan–Meier survival analysis was performed with failures before 1 month excluded, as specified in the study protocol. Sustained failure was defined as meeting failure criteria (IOP > 18 mmHg, <20% reduction from baseline, IOP ≤ 6 mmHg on two consecutive visits, loss of >2 lines of VA, or additional glaucoma surgery) at two consecutive available postoperative visits. The date of failure was recorded as the date of the second consecutive failing visit. Eyes not meeting sustained failure criteria were censored at their last available follow-up visit.

Of 70 eyes, 15 (21.4%) met sustained failure criteria during the follow-up period. The overall surgical survival was 100.0% at 1 month, 95.7% at 3 months, 90.0% at 6 months, 82.0% (95% CI: 71.4–94.1) at 12 months, and 75.3% (95% CI: 61.9–91.7) at 36 months.

When stratified by surgery type, the standalone surgery group (n = 22) had nine failures (40.9%), and the combined surgery group (n = 48) had six failures (12.5%). Survival in the standalone group was 86.4% at 3 months, 65.3% at 12 months, and 53.4% (95% CI: 24.1–100.0) at 36 months. Survival in the combined group was 100.0% at 3 months, 89.4% at 12 months, and 86.0% (95% CI: 74.4–99.4) at 36 months. The difference between groups was statistically significant (log-rank χ^2^ = 6.815, *p* = 0.009), with the combined surgery group demonstrating superior survival throughout the follow-up period.

#### 3.4.2. Complete Success Survival

**Figure 8:**
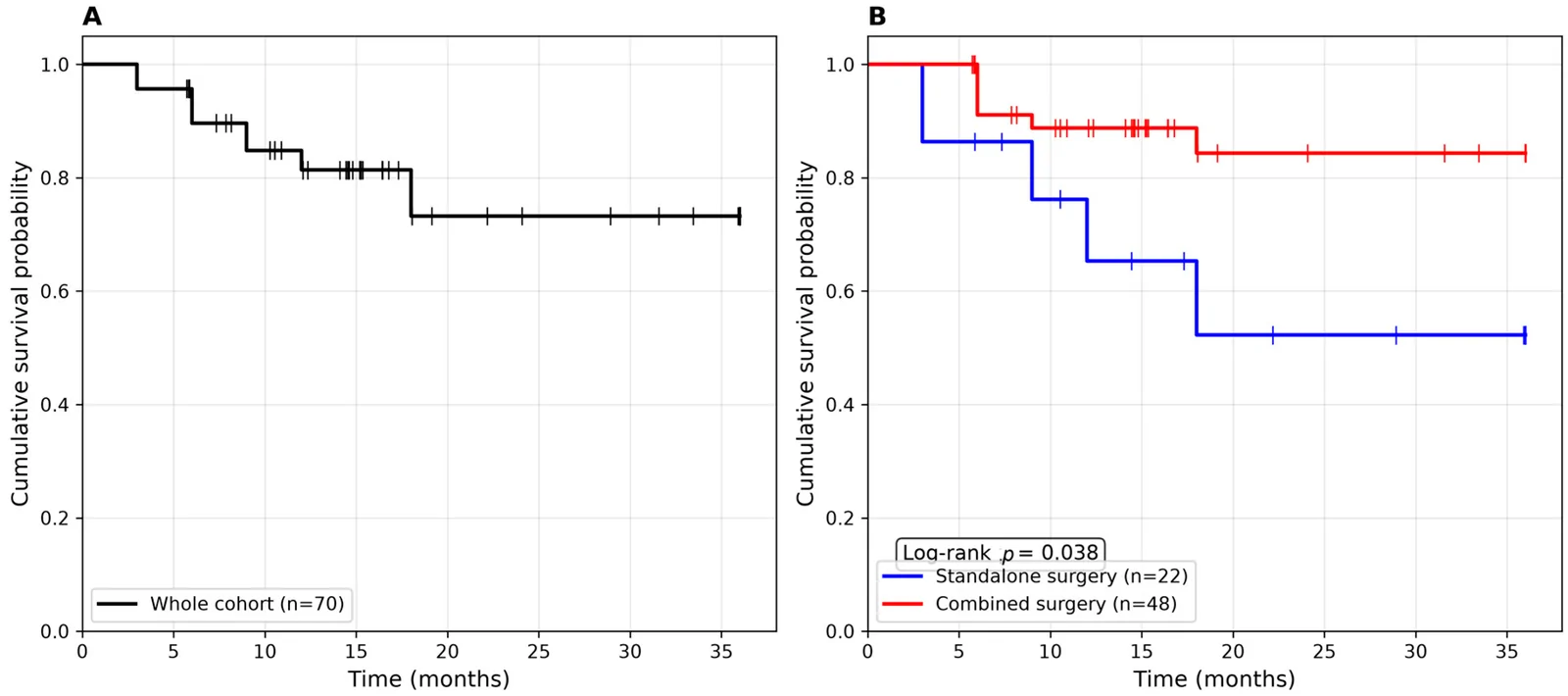
Kaplan–Meier cumulative survival curves for surgical success. (**A**) Whole cohort (n = 70). (**B**) Stratified by surgery type: standalone surgery (blue, n = 22) and combined phacoemulsification with glaucoma surgery (red, n = 48). Sustained failure was defined as meeting failure criteria (IOP > 18 mmHg, <20% reduction from baseline IOP, IOP ≤ 6 mmHg on two consecutive visits, loss of >2 lines of VA, or additional glaucoma surgery) at two consecutive available postoperative visits. Failures occurring before 1 month were excluded. The date of failure was recorded as the date of the second consecutive failing visit. Eyes not meeting sustained failure criteria were censored at their last available follow-up visit (tick marks). The difference between surgery groups was statistically significant (log-rank χ^2^ = 4.300, *p* = 0.038). Overall surgical survival was 100.0% at 1 month, 95.7% at 3 months, 89.6% at 6 months, 81.4% at 12 months, and 73.2% at 36 months. Survival at 36 months was 52.3% for the standalone group and 84.3% for the combined group.

When the definition of success was restricted to complete success (requiring IOP ≤ 18 mmHg, ≥20% reduction from baseline, and medications ≤ preoperative regimen), 18 eyes (25.7%) met failure criteria. The complete success survival was 100.0% at 1 month, 87.1% at 6 months, 79.1% (95% CI: 67.7–92.5) at 12 months, and 68.5% (95% CI: 51.8–90.7) at 36 months. The standalone group had nine failures and the combined group had nine failures, with survival at 36 months of 53.4% and 75.4%, respectively. The difference between groups did not reach statistical significance (log-rank χ^2^ = 4.300, *p* = 0.038) (Figure 8).

#### 3.4.3. Failure Characteristics

Among the 15 eyes meeting sustained failure criteria, 11 failures (73.3%) were due to inadequate IOP control (IOP > 18 mmHg or <20% reduction from baseline), three (20.0%) were due to loss of >2 lines of visual acuity, and one (6.7%) had both IOP and VA criteria met concurrently. The median time to failure was 9 months (range: 3–18 months). No eyes met the hypotony criterion (IOP ≤ 6 mmHg on two consecutive visits).

### 3.5. Safety

#### 3.5.1. Intraoperative Challenges

Most intraoperative difficulties were related to concomitant cataract surgery in eyes with intumescent cataracts or pseudoexfoliation syndrome. In these cases, pupils were rigid and resistant to dilation, necessitating the use of additional pupil-dilating devices and maneuvers. In all eyes with prior failed filtration surgery, dissection of the conjunctival and scleral flaps was technically challenging due to dense scarring. Notably, placement of the BCI into the suprachoroidal tunnel was uneventful in all cases, with no technical difficulties encountered.

#### 3.5.2. Postoperative Adverse Events

During the follow-up period, two notable postoperative events were observed. One patient developed a spontaneous retinal hemorrhage unrelated to the surgical procedure; the hemorrhage resolved spontaneously within one month, with full restoration of vision.

In another case, oozing from the SC occurred after LT, leading to hyphema. The hyphema resolved spontaneously within the first postoperative week. This patient had a history of diabetes mellitus.

Importantly, no cases of hypotony, shallow AC, or choroidal effusion were observed during the follow-up period.

### 3.6. LT: Analysis of Efficacy, Predictors, and Association with CLVs

LT was performed as an enhancement procedure when postoperative IOP exceeded the target threshold. LT was performed in 51 of 70 eyes (72.9%). The decision to perform LT was based on elevated postoperative IOP. The procedure involved laser application to create or enhance filtration through the surgical site.

LT was performed in 51 of 70 eyes (72.9%). The median time from surgery to first LT was 0.39 months (mean 0.92 months, range 0.03–8.32). The majority of procedures were performed early: 50.0% of all eyes underwent LT within 2 weeks, 61.4% within 1 month, and 71.4% within 6 months. All LT procedures in the study cohort were completed within 12 months of surgery.

Among 49 eyes with paired IOP measurements, LT achieved a significant reduction in IOP from a mean of 24.0 ± 5.4 mmHg to 12.8 ± 5.3 mmHg (mean reduction: 11.2 mmHg, 46.6%; paired *t*-test: t = 10.65, *p* < 0.001; and Wilcoxon: *p* < 0.001). The median IOP decreased from 23.0 to 12.0 mmHg. An IOP reduction of ≥30% was achieved in 69.4% of eyes, ≥50% in 44.9%, and IOP ≤18 mmHg was attained in 85.7% of eyes following the procedure.

Among the 51 eyes that underwent LT, 36 (70.6%) required a single session, 11 (21.6%) required two sessions, two (3.9%) required three, and two (3.9%) required four sessions. Eyes requiring multiple LT sessions had a higher mean pre-procedure IOP (26.9 mmHg) compared to those requiring a single session (22.7 mmHg), though this difference was not statistically significant (Mann–Whitney *p* = 0.339). IOP reduction was comparable between groups (single: 10.5 mmHg; multiple: 12.8 mmHg; and *p* = 0.339). No significant differences in baseline characteristics (age, severity, surgical group, baseline IOP, and baseline medications) were observed between eyes requiring single vs. multiple LT sessions.

Baseline IOP was the only significant univariate predictor of IOP reduction after LT (Pearson r = 0.69, *p* < 0.001; Spearman rs = 0.68, *p* < 0.001)—eyes with higher pre-procedure IOP experienced greater absolute reduction. A trend was observed for sex (male: median reduction 13.5 mmHg vs. female: 9.0 mmHg, *p* = 0.084). Age, glaucoma type, severity, prior surgery, pseudophakic status, surgical group, and time to first LT were not significantly associated with IOP reduction.

**Figure 9:**
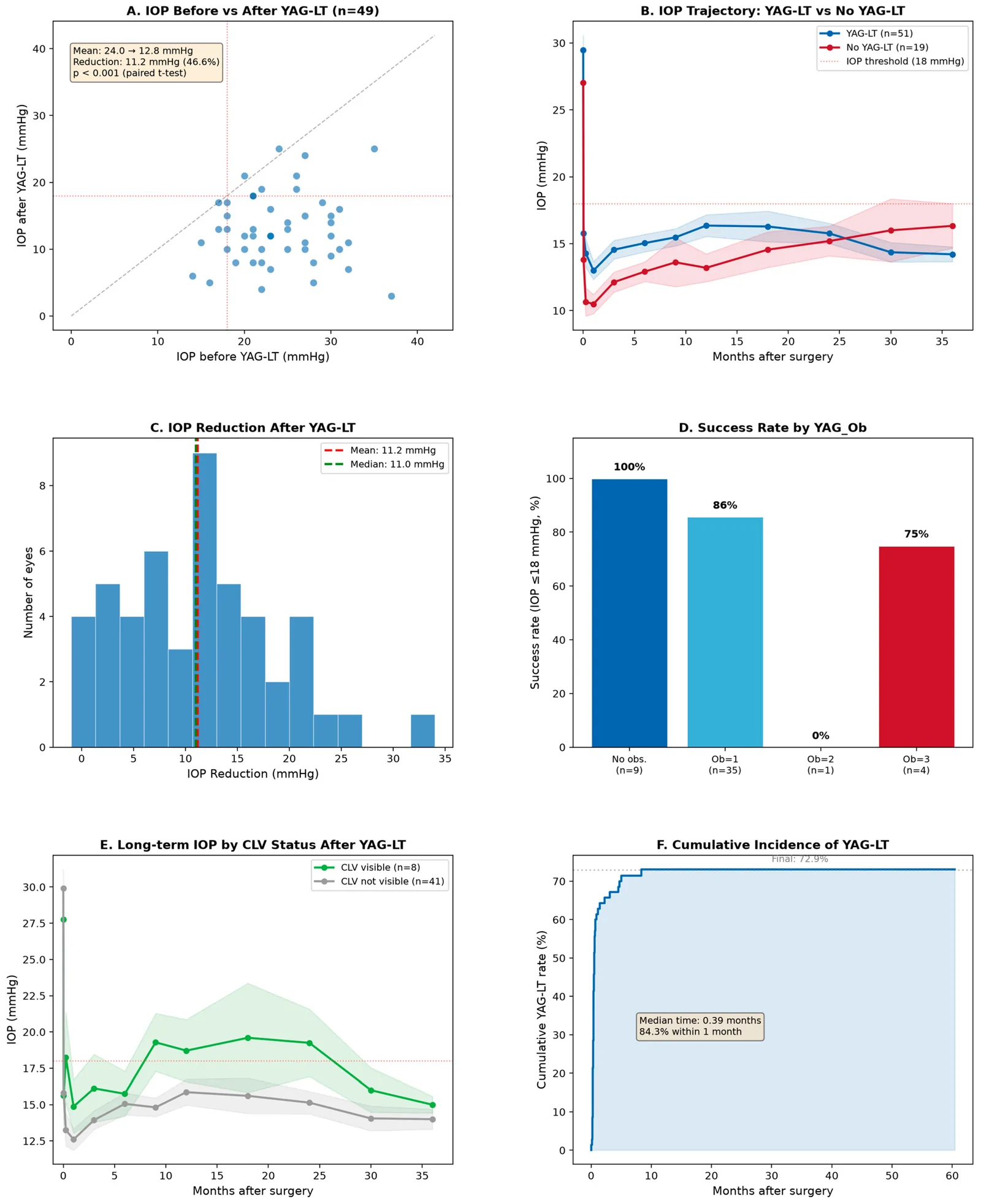
LT analysis. (**A**) Paired IOP before vs. after LT (n = 49 eyes, *p* < 0.001). Dashed line = unity; red dotted lines = 18 mmHg threshold. (**B**) Long-term IOP trajectory: LT vs. no LT groups (mean ± SE). (**C**) Distribution of IOP reduction after LT. (**D**) Success rate (IOP ≤18 mmHg) by obstruction grade (YAG_Ob). (**E**) Long-term IOP trajectory by CLV status after LT (mean ± SE). The red dashed line indicates 18 mmHg. (**F**) Cumulative incidence of LT over follow-up (Kaplan–Meier estimate).

To assess whether LT status influenced long-term outcomes, the following two linear mixed-effects models with random intercept per eye were fitted:

Model 1 (IOP trajectory): The main effect of LT was not statistically significant (β = 2.527, SE = 1.696, and *p* = 0.136), indicating no significant difference in overall IOP level between eyes that underwent LT and those that did not. The LT × time interaction was also non-significant (β = −0.083, *p* = 0.318), suggesting parallel IOP trajectories. Time, age, severity, and surgical group were not significant predictors. The ICC was 0.333, indicating moderate between-eye variability (Figure 9).

Model 2 (Medication trajectory): The LT × time interaction was significant (β = 0.029, SE = 0.014, and *p* = 0.035), indicating that eyes undergoing LT had a gradual increase in medication count over time relative to eyes without LT. The main effect of LT was not significant (β = −0.161, *p* = 0.571), and time showed a significant negative trend in the no-YAG group (β = −0.029, *p* = 0.022). This suggests that while both groups started with similar medication counts, the LT group required progressively more medications over follow-up.

Among 49 eyes with CLV data after LT, CLVs were visible in eight eyes (16.3%) and not visible in 41 eyes (83.7%). IOP after LT was comparable between groups (CLVs visible: 12.8 ± 5.6 mmHg; CLVs not visible: 12.9 ± 5.3 mmHg; and Mann–Whitney *p* = 0.935). IOP reduction was also similar (CLVs visible: 9.5 mmHg; CLVs not visible: 11.5 mmHg; and *p* = 0.464). A mixed-effects model examining the association between CLVs visibility after LT and long-term IOP (postoperative trajectory) found no significant effect (β = 1.680, SE = 1.852, and *p* = 0.365), adjusted for time, age, severity, and surgical group. Time was the only significant predictor (β = 0.062, *p* = 0.033), reflecting a slight upward drift in IOP over follow-up.

At the time of laser application fluid movement was observed in 85.7% eyes (30/35), and hemorrhage from SC was observed in three eyes. In one eye some hemorrhage occurred from iris tissue.

The results show that LT, which was performed in 72.9% of eyes, was most commonly performed within the first month (median 0.39 months), as an early enhancement procedure. LT achieved a substantial and statistically significant IOP reduction of 11.2 mmHg (46.6%), with 85.7% of eyes achieving IOP ≤ 18 mmHg. Baseline IOP was the strongest predictor of absolute IOP reduction (r = 0.69, *p* < 0.001)—higher pre-procedure IOP yielded greater reduction. A total of 29.4% of eyes required multiple LT sessions, but no baseline characteristics distinguished single vs. multiple procedures. LT status did not significantly affect long-term IOP trajectory (*p* = 0.136) but was associated with a gradual increase in medication burden over time (YAG × time interaction, *p* = 0.035), suggesting that eyes requiring LT represent a more challenging subgroup. CLV visibility after LT was infrequent (16.3%) and was not associated with post-procedure IOP, IOP reduction, or long-term IOP trajectory (*p* = 0.365).

### 3.7. Biomicroscopic Visualization and Morphological Characteristics of CLVs After Surgery

In the early postoperative period (first few days), mild conjunctival swelling at the surgical site was observed in all cases, consistent with expected surgical trauma.

Bleb formation was uncommon: a transient bleb was noted in only one case that resolved spontaneously within one week. No other blebs were identified during the follow-up period. CLVs were visible on slit-lamp examination in 34 of 70 eyes (48.6%). The superior nasal quadrant was the most frequent location (46 observations), followed by the superior temporal quadrant (17 observations) (Table 4).

Following surgery, the proportion of eyes with visible CLVs dropped sharply to 14.3% at day 1 postoperatively, followed by a transient increase to 30.0% at 1 month, and then a progressive decline to 0% at 36 months.

**Figure 10:**
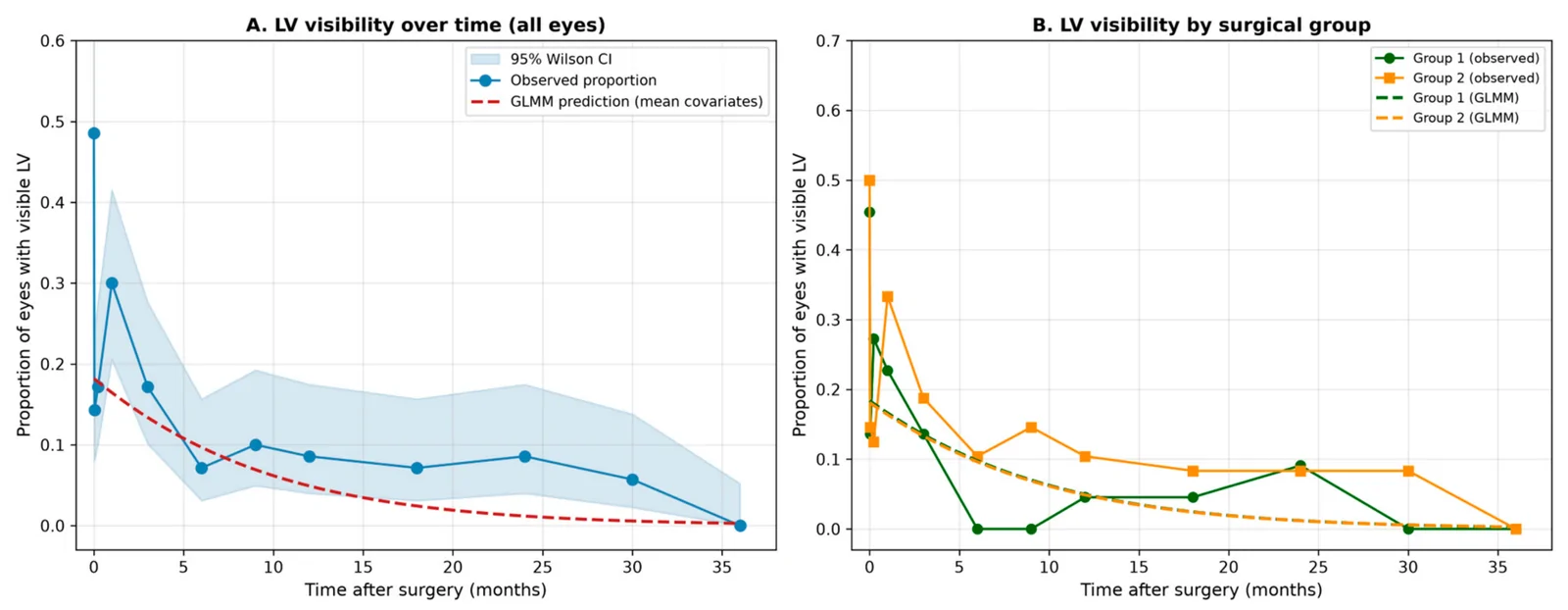
CLVs visibility on slit-lamp examination over the 36-month follow-up period. (**A**) Overall proportion of eyes with visible CLVs at each time point (blue circles) with 95% Wilson confidence intervals (shaded area) and GLMM-predicted probability (dashed red line). (**B**) CLV visibility by surgical group, with observed proportions (circles/squares) and GLMM-predicted curves (dashed lines) for standalone surgery group (green) and combined surgery group (orange).

A total of 840 observations from 70 eyes were included in the GLMM. The results of the GLMM show that time after surgery was the strongest predictor of CLVs visibility: each additional month of follow-up was associated with an 11.5% reduction in the odds of CLVs being visible (OR = 0.885, 95% CI 0.849–0.923, and *p* < 0.001). Age was also a significant predictor, with each additional year of age reducing the odds of CLV visibility by 7.5% (OR = 0.925, 95% CI 0.865–0.990, and *p* = 0.025). Surgical group, LT status, and glaucoma severity were not significantly associated with CLV visibility (all *p* > 0.05) (Figure 10).

The random intercept variance was estimated at 2.90 (SD = 1.70), yielding an ICC of 0.47. This indicates that approximately 47% of the total latent variance in CLV visibility was attributable to between-eye differences, substantiating the use of a mixed-effects model to account for within-eye correlation.

#### 3.7.1. Association Between CLV Visibility and IOP: A Linear Mixed-Effects Analysis

A total of 664 observations from 70 eyes were available for analysis. CLVs were visible on slit-lamp examination in 122 observations (18.4%) and not visible in 542 observations (81.6%). The mean IOP across all visits was 15.95 mmHg (SD 7.40, range 1–57). When LVs were visible, mean IOP was 17.40 mmHg compared to 15.63 mmHg when LVs were not visible.

In the linear mixed-effects model, CLV visibility was significantly associated with higher IOP. Eyes with visible CLVs had IOP 2.40 mmHg higher on average than eyes without visible LV (β = 2.395, SE = 0.790, 95% CI: 0.846–3.943, z = 3.03, and *p* = 0.002), after adjusting for time, age, surgical group, glaucoma severity, and LT. LT was also a significant predictor: eyes without LT had IOP 2.04 mmHg lower than those with perforation (β = −2.039, *p* = 0.030). Time showed a trend toward reduction in IOP (β = −0.055 per month, *p* = 0.061). Age, surgical group, and glaucoma severity were not significantly associated with IOP.

The random intercept variance was 6.03 (SD = 2.46), with a residual variance of 46.43 (SD = 6.81), yielding an ICC of 0.115. This indicates that approximately 12% of the total variance in IOP was attributable to between-eye differences, confirming the need for a mixed-model approach.

CLV visibility on slit-lamp examination is concurrently associated with higher IOP (approximately 2.4 mmHg higher). However, the lagged analysis indicates that CLVs visibility does not predict future IOP elevation, suggesting that the observed association may reflect a shared underlying mechanism rather than a direct causal effect of CLVs visibility on IOP.

#### 3.7.2. Morphological Characteristics of CLVs After Surgery

**Figure 11:**
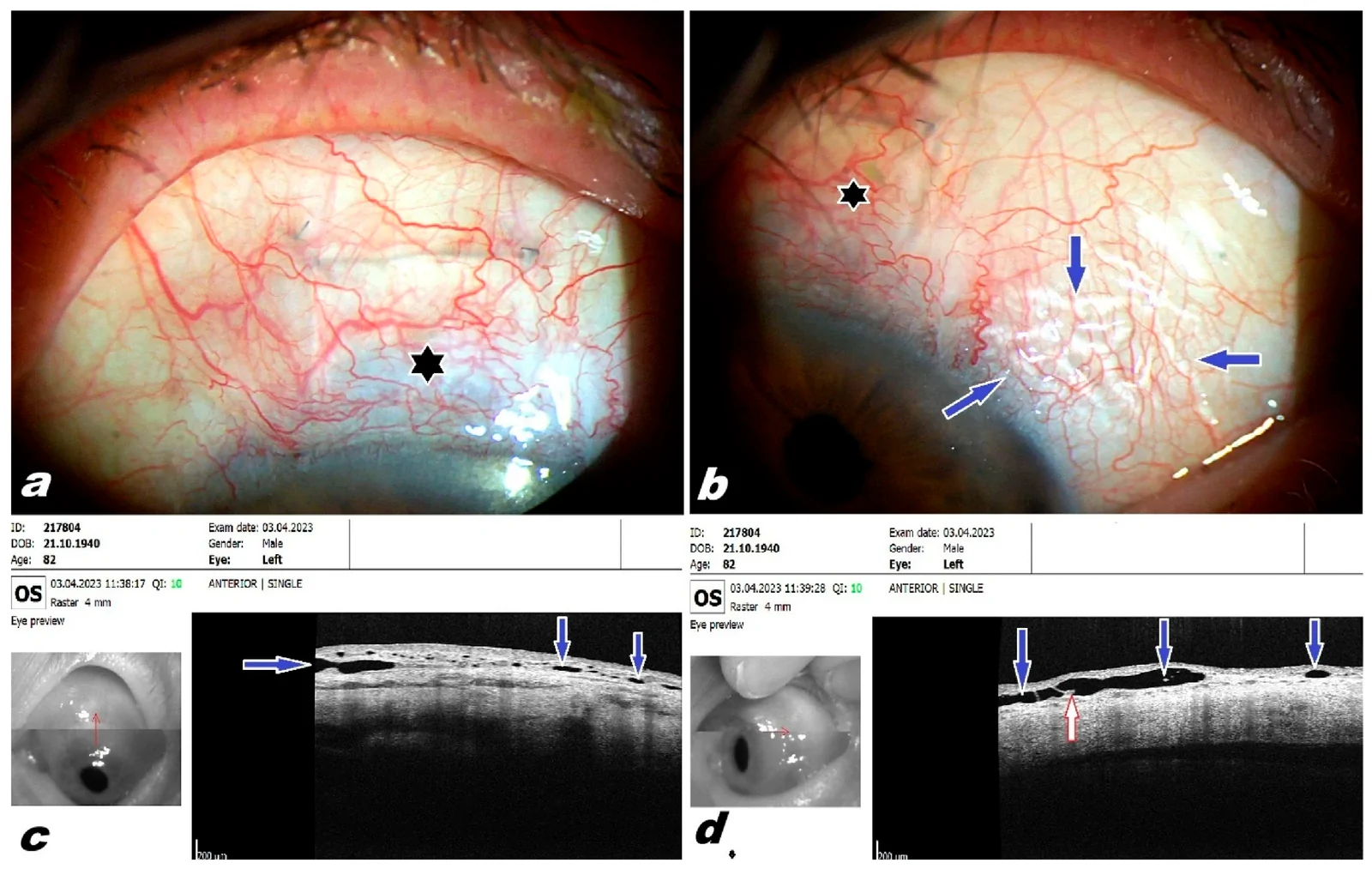
Biomicroscopic visualization of CLVs and OCT confirmation of their lymphatic nature. Slit-lamp anterior views of the surgical site and superior temporal quadrant of the conjunctiva in the left eye, and corresponding OCT scans of these areas, obtained 3 months after surgery. (**a**) At the surgical site (marked by a black star with white borders), no CLVs are visible. (**b**) A plexus of CLVs (indicated by indigo arrows with white borders) arises directly from the sclera in the superior temporal quadrant; no visible connection with the surgical site is noted. (**c**) OCT scan of the surgical site shows CLVs (indigo arrows with white borders) that were not visible on biomicroscopy. The red arrow indicates the OCT scan direction. (**d**) OCT scan of a conjunctival area with biomicroscopically visible CLVs demonstrates lymphatic vessels (indigo arrows with white borders) with valve-like formations in their lumens (white arrow with red borders).

**Figure 12:**
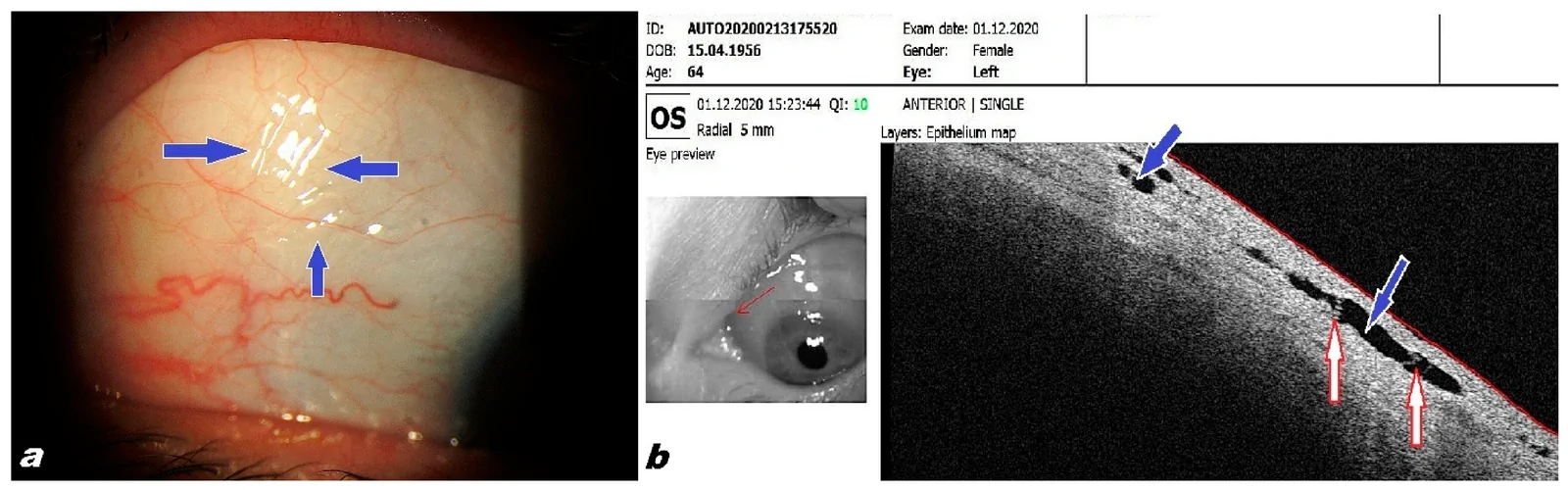
Biomicroscopic visualization of CLVs and OCT confirmation of their lymphatic nature (another case). Left eye of a 69-year-old female patient, 1 month after surgery. (**a**) Slit-lamp anterior view of the superior medial quadrant of the conjunctiva, showing a plexus of transparent CLVs (indicated by indigo arrows with white borders) arising directly from the sclera at a location distant from the surgical site. (**b**) Corresponding OCT scan of the area demonstrates CLVs (indigo arrows) with intracavitary flap-like structures consistent with valve-like formations in their lumens (white arrows with red borders). The red arrow indicates the OCT scan direction.

The lymphatic nature of the transparent vessels was supported by OCT findings in all observed cases. OCT imaging revealed valve-like formations within the vessel lumina (Figure 11a–d and Figure 12a,b).

**Figure 13:**
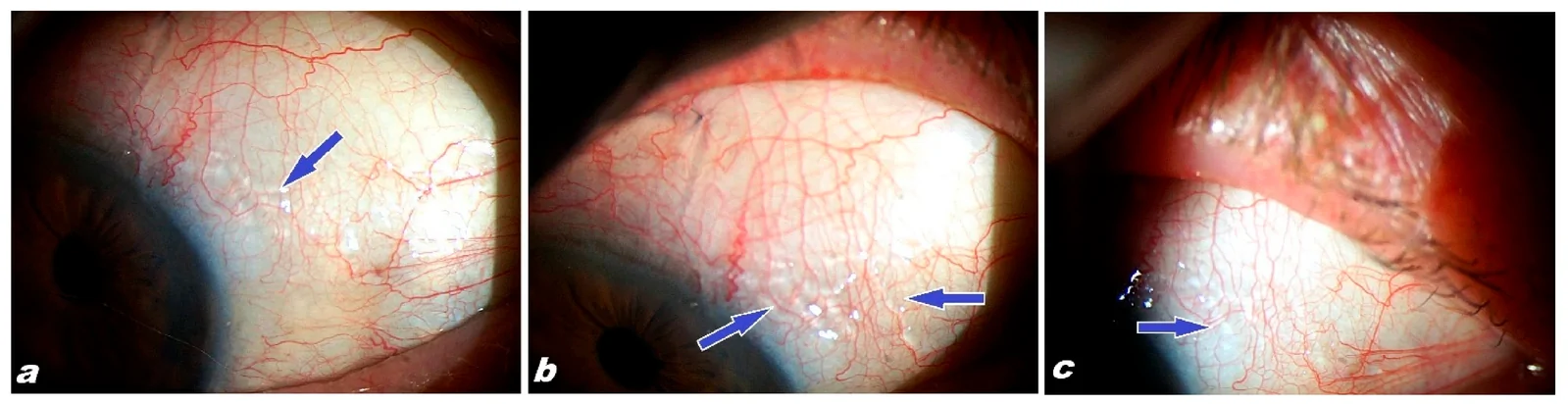
Visualization of CLVs by temporary mechanical obstruction of lymphatic outflow via upper lid margin displacement. Left eye of an 82-year-old male patient, 3 months after surgery; IOP 8 mmHg without IOP-lowering medication. (**a**) Slit-lamp anterior view of the superior temporal quadrant of the bulbar conjunctiva shows transparent CLVs (indicated by indigo arrows with white borders) near the limbus. The vessels arise directly from the sclera, show no visible connection with the surgical site, and run parallel to the limbus. (**b**) CLVs become more prominent when lymphatic outflow is transiently obstructed by the upper lid margin, increasing vessel filling and visibility (indigo arrows with white borders). The photograph was captured from a video clip during the maneuver; the lid margin was momentarily withdrawn to acquire the image. (**c**) Rapid change in CLV appearance occurs immediately after release of pressure by lifting the lid margin, demonstrating the dynamic, flow-dependent nature of vessel visibility.

**Figure 14:**
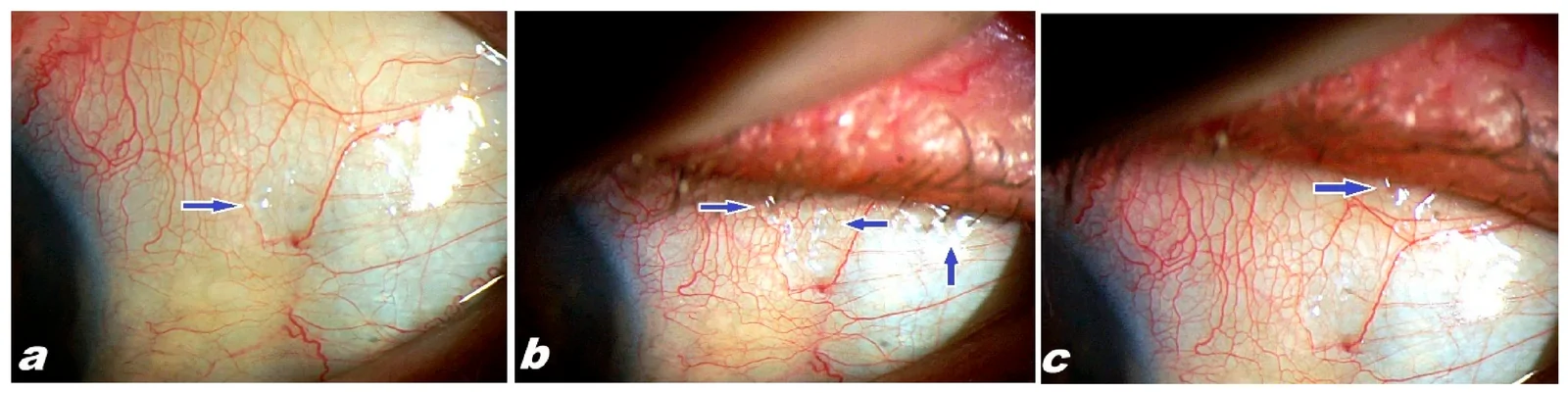
Visualization of CLVs by temporary mechanical obstruction of lymphatic outflow via upper lid margin displacement (another case). Slit-lamp anterior view of the superior bulbar conjunctiva of the left eye, 2 years after surgery. (**a**) In the superior temporal quadrant, a single small CLV is visible at baseline (indicated by an indigo arrow with white borders). (**b**) Upon transient obstruction of lymphatic outflow by the upper lid margin, CLVs become markedly more prominent due to increased intraluminal filling (indigo arrows with white borders). (**c**) As soon as pressure is released by lifting the lid margin, the CLVs rapidly become less prominent, demonstrating their flow-dependent visibility.

**Figure 15:**
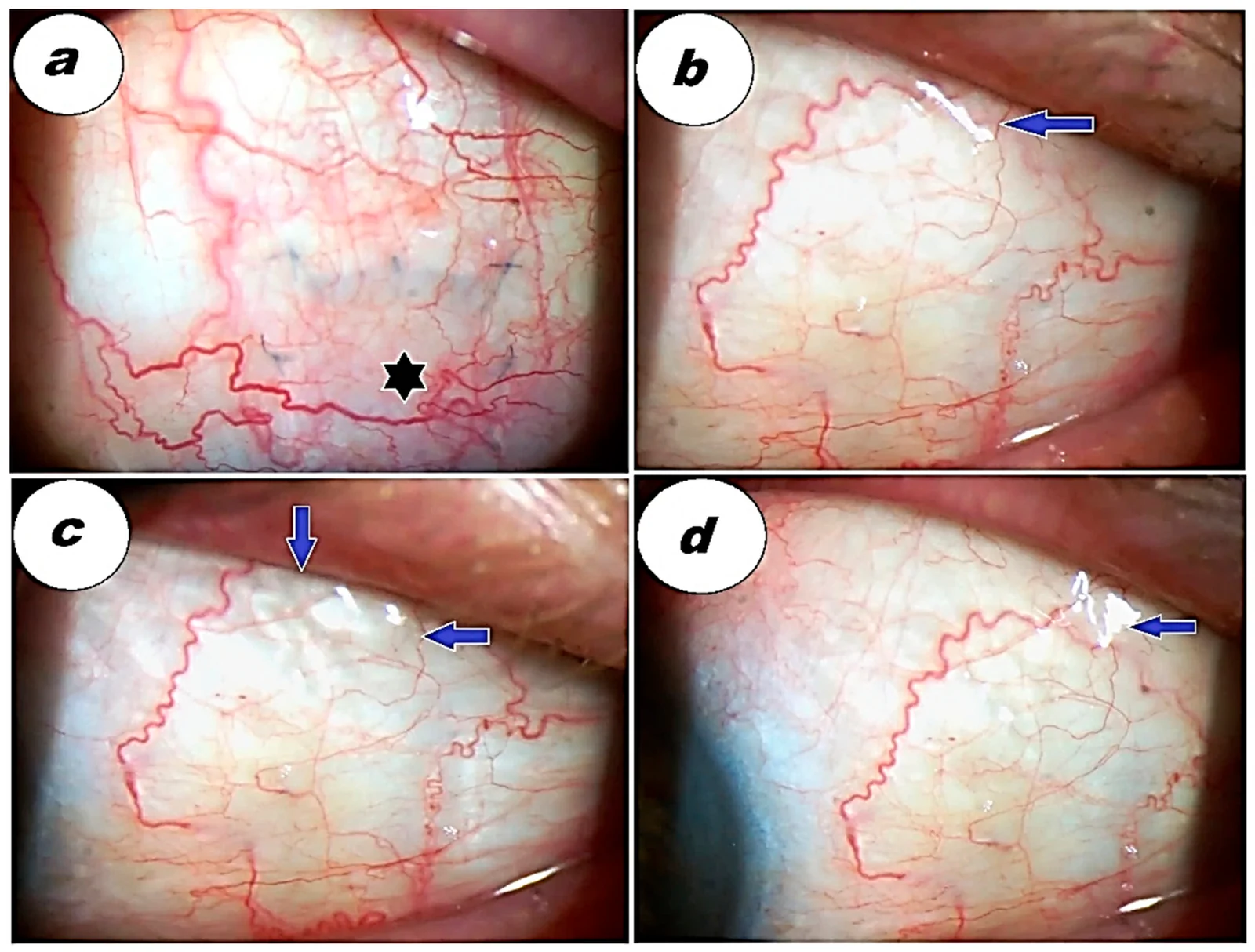
Visualization of CLVs by temporary mechanical obstruction of lymphatic outflow via upper lid margin displacement (another case). Left eye of an 88-year-old male patient, 15 days after surgery. (**a**) Slit-lamp anterior view of the surgical site (marked by a black star with white borders) shows absence of blebs and CLVs, with mild postoperative edema. (**b**) In the superior temporal quadrant, a single transparent CLV becomes visible as transient pressure is applied via lid margin movement (indigo arrow with white borders). (**c**) Sweeping movements of the lid margin over the conjunctiva induce the appearance of a CLV plexus (indigo arrows with white borders), likely due to increased intraluminal filling. (**d**) As soon as lid movements cease, the CLVs rapidly become less prominent, confirming their flow-dependent visibility.

Gentle manipulation of the upper eyelid margin over the conjunctiva facilitated detection of otherwise inconspicuous transparent vessels, as demonstrated in Figure 13a–c, Figure 14a–c and Figure 15a–d (see Supplementary Material, Video S4).

**Figure 16:**
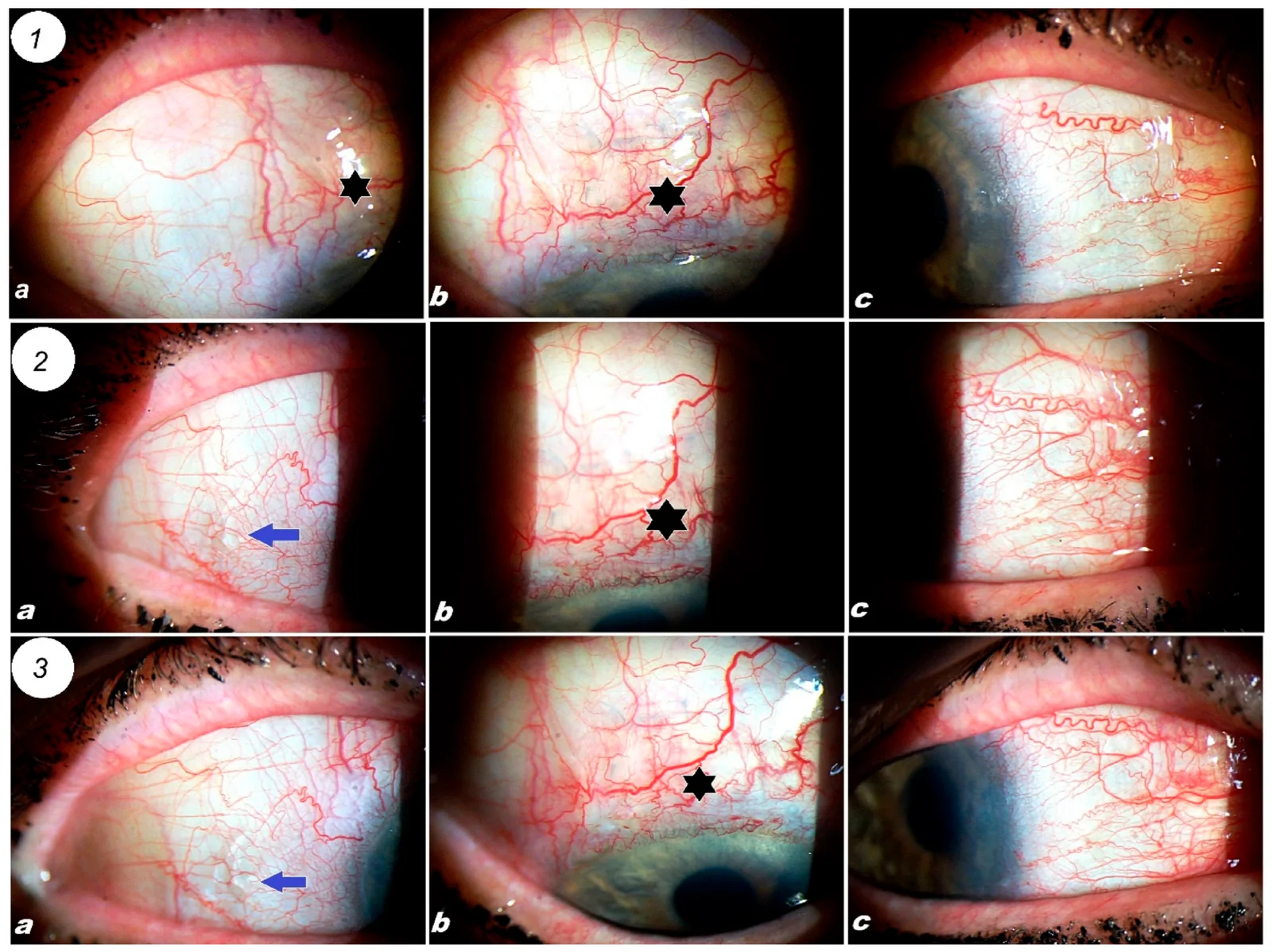
Appearance of CLVs in the temporal quadrant of the superior bulbar conjunctiva after LT in a pseudophakic right eye. Seventy-four-year-old female patient, 2 weeks after glaucoma surgery. Panel 1 (baseline, before LT). Slit-lamp anterior views: (**1a**) Superior temporal quadrant. (**1b**) Surgical site. (**1c**) Superior medial quadrant. No CLVs are visible in any of these areas at baseline. Panel 2 (immediately after LT). (**2a**) A transparent CLV is observed arising directly from the sclera in the superior temporal quadrant (indicated by an indigo arrow with white borders). (**2b**,**c**) The surgical site and superior medial quadrant show no visible changes compared to baseline. Panel 3 (10 min after LT). (**3a**) CLVs in the superior temporal quadrant are more prominent at the same location as in Panel 2a, suggesting increased intraluminal filling. (**3b,c**) The surgical site and superior medial quadrant remain unchanged. The black star with white borders indicate the surgical site.

Among the 51 eyes undergoing LT, CLVs were observed immediately post-procedure in six eyes (Figure 16(1a–c,2a–c,3a–c); see Supplementary Material: Video S5). In the remaining eyes, CLVs appeared at later time points.

**Figure 17:**
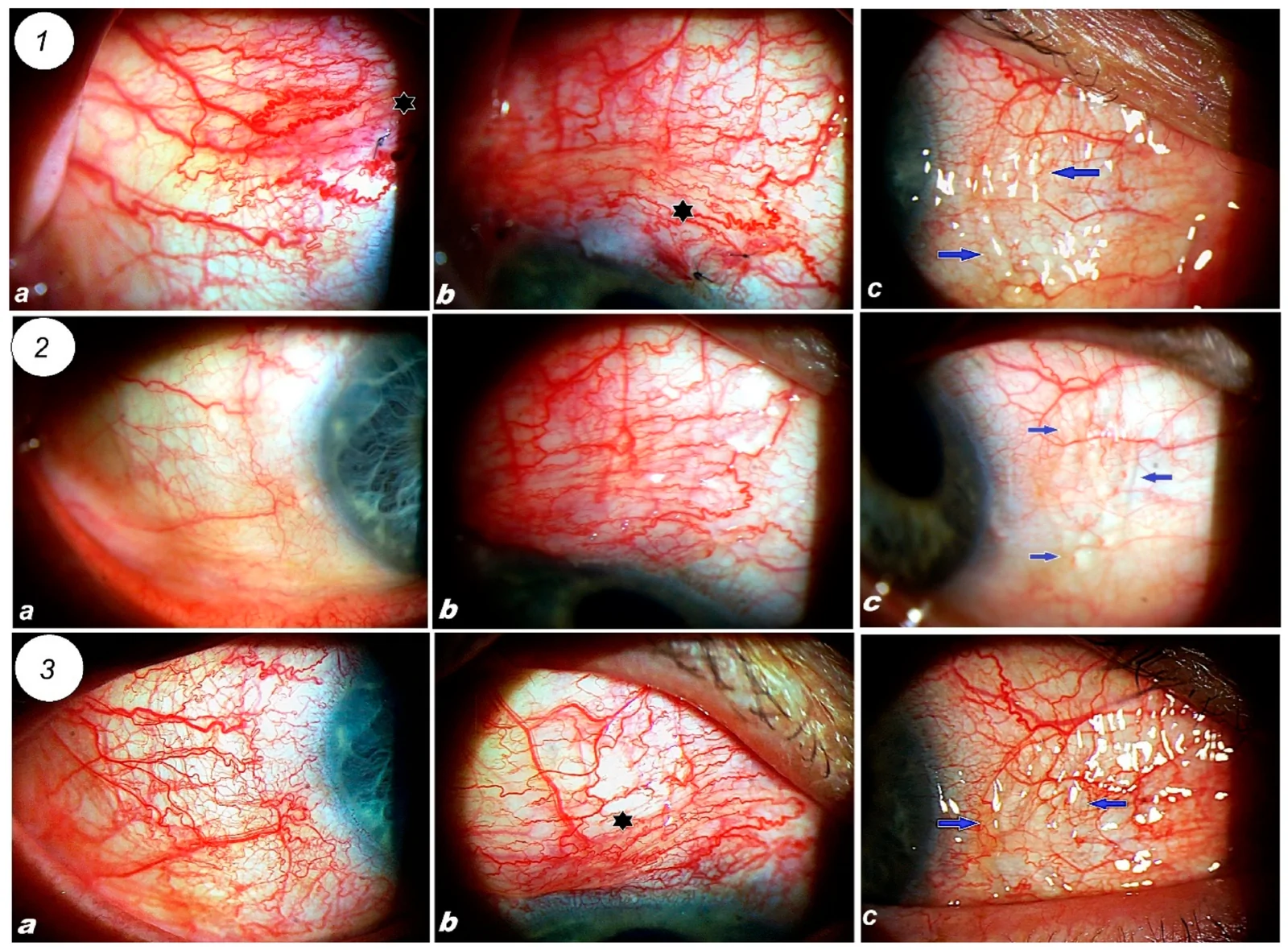
Postoperative visualization of CLVs without LT and their site-specific reappearance at the same location after LT. Slit-lamp anterior views of the right eye of a 61-year-old male patient. Panel 1 (1 week after surgery; IOP 16 mm Hg on 3 classes of IOP-lowering medication). (**1a**) Superior temporal quadrant: no CLVs are visible. (**1b**) Surgical site (marked by a black star with white borders): absence of blebs and CLVs. (**1c**) Superior medial quadrant and area of medial rectus muscle insertion: 2–3 prominent CLVs (indicated by indigo arrows with white borders) run parallel to the limbus and arise directly from the sclera near the medial rectus insertion. Panel 2 (2 weeks after surgery, prior to LT; IOP 22 mm Hg on 3 classes of IOP-lowering medication). (**2a**,**b**) Superior temporal quadrant and surgical site show no changes compared to Panel 1. (**2c**) In the superior nasal quadrant, the previously observed CLVs are reduced in size (indigo arrows with white borders), indicating a diminishing lymphatic signal over time. Panel 3 (1 week after LT). (**3a,b**) Superior temporal quadrant and surgical site remain free of CLVs and blebs. (**3c**) At the identical anatomical location as in Panel 1c, a well-developed plexus of prominent CLVs is observed, bulging with transparent fluid (indigo arrows with white borders). The vessels arise directly from the sclera, confirming site-specific recurrence after LT.

In eyes where CLVs became visible after initial surgery (without LT) and subsequently disappeared over time, CLVs were observed to reappear at the same anatomical location following LT. This recurrent, site-specific pattern is demonstrated in Figure 17.

**Figure 18:**
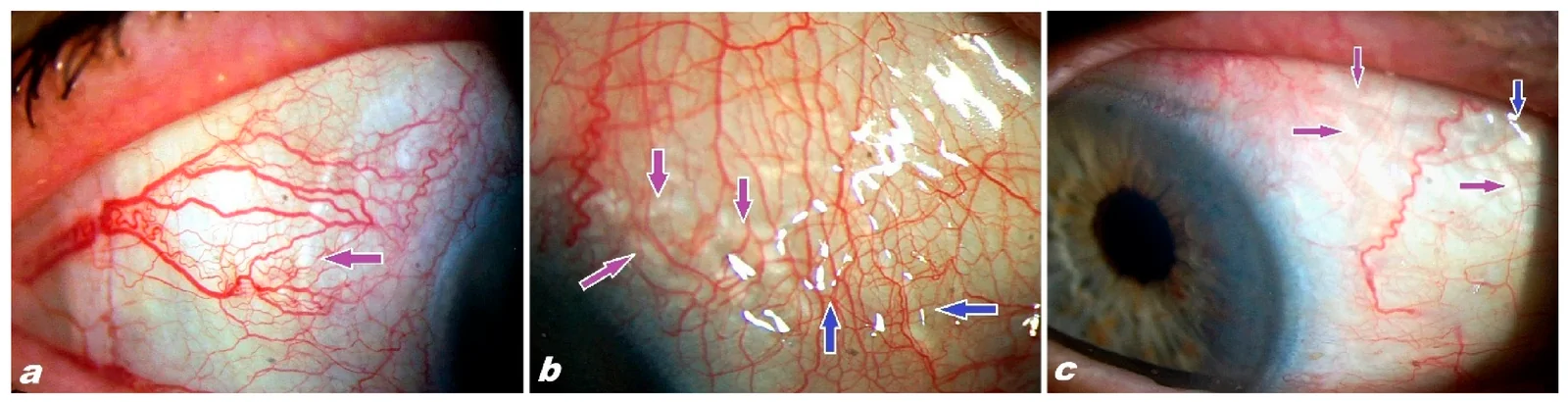
Slit-lamp anterior views of the superior conjunctiva in three different patients who underwent LT for elevated postoperative IOP, demonstrating development of CLVs in distinct conjunctival layers, with no anatomical relationship to conjunctival blood vessels. (**a**) Development of a deep CLV (indicated by a lilac arrow with white borders), running parallel to the limbus at a measurable distance from it. The vessel has a dull appearance; conjunctival blood vessels are observed coursing over its surface, confirming its deeper anatomical plane. (**b**) Formation of a plexus comprising both superficial and deep CLVs. Superficial CLVs (indigo arrows with white borders) exhibit sharp contours, prominent luminal filling, and a bright appearance; conjunctival blood vessels run beneath them. Deep CLVs (lilac arrows with white borders) display blurred contours, a duller appearance and color, and are less conspicuous. Notably, superficial CLVs appear to arise from and represent a continuation of deeper vessels, indicating a hierarchical lymphatic architecture. (**c**) View of the superior temporal quadrant (adapted from Figure 15c), showing a plexus of transparent superficial (indigo arrows with white borders) and deep (lilac arrows with white borders) CLVs with morphological features consistent with those observed in Figure 18b.

CLVs were frequently observed to originate directly from the sclera in regions distant from the surgical site, with no visible anatomical connection to it. This pattern is illustrated in Figure 11b, Figure 12a, Figure 16(2a,3a), Figure 17(1c,2c,3c) and Figure 18a.

No consistent spatial pattern of CLV appearance was identified. CLVs were observed running parallel to the limbus in some instances, while in others they oriented perpendicularly to the limbal margin. A plexiform arrangement was noted in several cases (Figure 13a–c and Figure 15c,d), whereas in other observations CLVs remained as isolated single or paired vessels (see Supplementary Material: Video S6).

In a subset of eyes undergoing LT at elevated IOP, distinct layers of CLVs could be identified—namely, superficial and deep CLVs—either immediately after the procedure or within a short post-procedural interval. This stratification is illustrated in Figure 18b, and the dynamic visibility of layered vessels is demonstrated in Supplementary Video S7.

### 3.8. OCT- Based Identification and Characterization of CLVs in the Postoperative Period

As part of the standard pre- and postoperative protocol, patients were scheduled for OCT imaging of the superior bulbar conjunctiva at week 1 and at months 1, 3, 6, 9, 12, 18, 24, 30, and 36. Preoperatively every patient had undergone OCT evaluation of to be the surgery site and nearby areas. In clinical practice, however, OCT could not be performed at every scheduled interval. Fourteen patients did not undergo any postoperative OCT due to logistical constraints.

**Figure 19:**
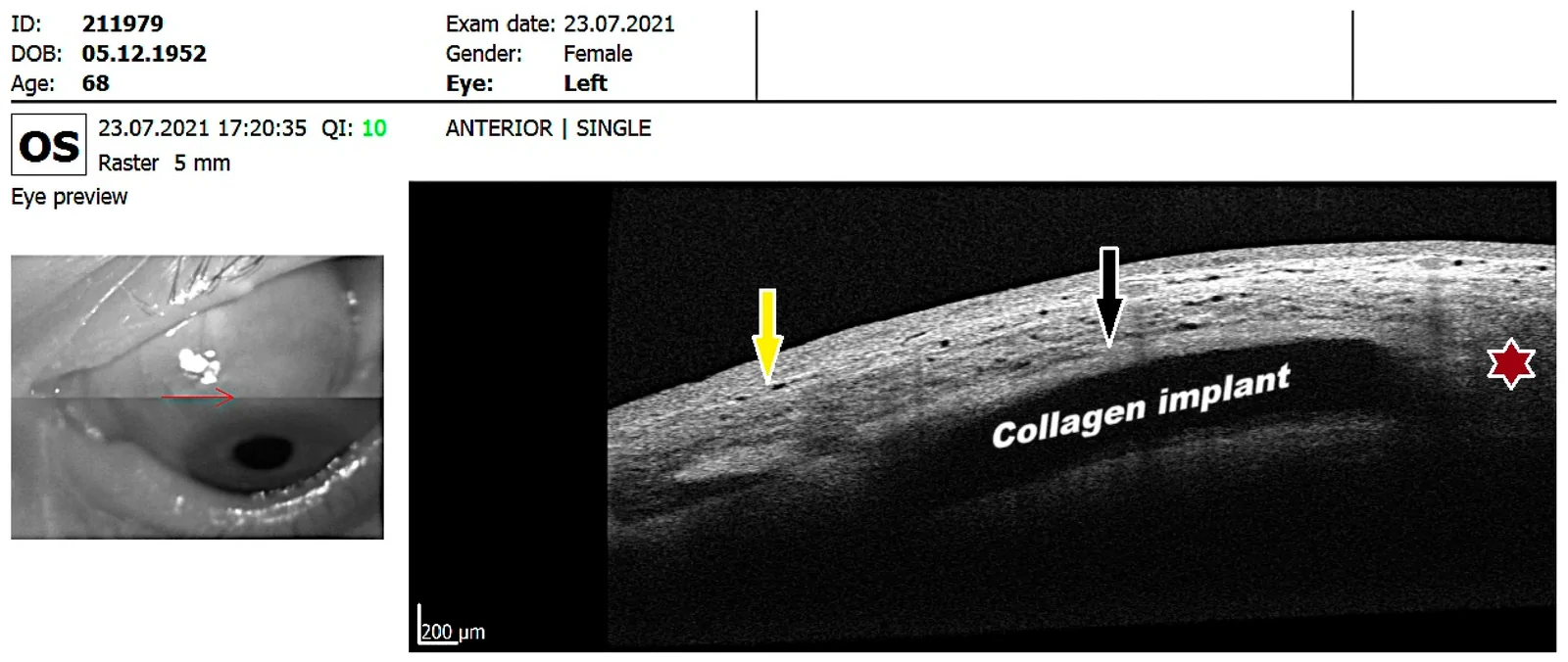
OCT imaging of the surgical site in the left eye of a 68-year-old female patient, 1 month after glaucoma surgery. (Left panel) Slit-lamp anterior view of the surgical area. The red arrow indicates the region subjected to OCT scanning. (Right panel) Corresponding OCT cross-sectional scan. No conventional filtration bleb is present within the scanned region. The collagen implant is covered by the superficial scleral flap (black arrow with white borders). The overlying conjunctiva is indicated by a yellow arrow with white borders. The sclera is marked by a red star with white borders. The red arrow indicates direction of OCT scan.

Before surgery, no eye had CLVs identified on OCT. After surgery, no formation of a conventional filtration bleb was observed in any of the study cases (Figure 19), indicating that AH flowed from the AC only to the SCS. In a single instance, OCT imaging revealed a transient bleb cavity that persisted for only one week.

The spatial distribution of OCT-identified CLVs demonstrated heterogeneous localization patterns across the superior bulbar conjunctiva. In 22.5% of eyes, CLVs were detected in all anatomical areas investigated. Simultaneous identification in the superior temporal and medial quadrants was observed in 17.2% of eyes. Isolated localization was noted in the superior medial quadrant in 16.8% of eyes and in the superior temporal quadrant in 10.5%. In 9.6% eyes, CLVs were identified exclusively at the surgical site.

Postoperatively, OCT was performed at the following 11 follow-up time points: 1 day, 1 week, 1 month, 3, 6, 9, 12, 18, 24, 30, and 36 months. A total of 215 observations from 57 eyes had both OCT identification of CLV data and IOP measurements available for analysis. The primary outcomes were (1) IOP measured at each follow-up visit, and (2) the number of glaucoma medications prescribed. IOP > 18 mmHg was considered as high IOP. The primary predictor was OCT identification of CLVs (binary: identified vs. not identified).

Among 57 eyes with postoperative OCT data, CLVs were identified in 188 of 215 observations (87.4%). Visibility was highest in the early postoperative period (100% at 1 day, 95.8% at 1 week, and 100% at 1 month) and gradually declined to 71.4% at 36 months (Table 1). No eye had LV identified on OCT before surgery.

In the primary LMM (215 observations, 57 eyes), OCT LV visibility was not significantly associated with IOP (β = −0.394 mmHg, 95% CI [−2.552, 1.764], and *p* = 0.720). Time (β = 0.074/month, *p* = 0.041), age (β = −0.169/year, *p* = 0.006), and absence of YAG perforation (β = −1.999, *p* = 0.037) were significant predictors of IOP. The random intercept variance was 3.09 (σ_u = 1.757) with a residual variance of 20.75 (σ_e = 4.555), yielding an ICC of 0.130.

In the logistic regression for IOP > 18 mmHg (32 events, 14.9%), OCT LV visibility was not significantly associated with high IOP (OR = 1.682, 95% CI [0.360, 7.855], and *p* = 0.508). Age (OR = 0.896 per year, *p* = 0.008) and surgical combined surgery group (OR = 0.311, *p* = 0.049) were significant predictors.

In the LMM for medication count, OCT LV visibility was not significantly associated with the number of prescribed medications (β = −0.268, 95% CI [−0.645, 0.109], and *p* = 0.163). Time was the only significant predictor (β = 0.019/month, *p* = 0.002), indicating a gradual increase in medications over follow-up. The ICC was 0.356, indicating substantial between-eye variability.

The lagged analysis (OCT identification of CLVs at visit t predicting IOP at visit t + 1) showed no significant association (β = −0.852, 95% CI [−2.830, 1.126], and *p* = 0.399), suggesting that OCT LV visibility does not predict future IOP. Similarly, the lagged model for medications was non-significant (β = −0.120, *p* = 0.513).

A significant interaction between OCT LV visibility and time was observed for IOP (β = 0.241, *p* = 0.026), indicating that the relationship between OCT LV visibility and IOP changes over the follow-up period. In the early postoperative phase (≤1 month), eyes with visible LV had lower IOP (13.2 vs. 15.0 mmHg), whereas beyond 12 months, the pattern reversed (16.0 vs. 15.1 mmHg). The interaction was not significant for medications (β = 0.032, *p* = 0.083).

The null association between OCT LV visibility and IOP was robust across all 11 sensitivity analyses, with the following two exceptions: (1) the LT: no subgroup (n = 51) showed OCT identification of CLVs associated with lower IOP (β = −3.149, *p* = 0.027), and (2) the OCT identification of LV × time interaction was significant (*p* = 0.026). For medications, the unadjusted model was significant (β = −0.489, *p* = 0.008), but adjustment for covariates attenuated the effect to non-significance (*p* = 0.163), suggesting confounding rather than a direct effect.

CLVs were identified on OCT in the vast majority of eyes after glaucoma surgery (87.4% of observations), despite being absent in all eyes preoperatively. However, OCT LV visibility was not independently associated with IOP or the number of prescribed medications in the primary adjusted models. The significant LV × time interaction for IOP suggests that the functional role of the CLVs evolves as the surgical site matures—with a protective (lower IOP) effect early on that diminishes over time. The lagged analysis confirmed the absence of a direct causal relationship. These findings were robust across multiple sensitivity analyses.

## 4. Case Reports

**Figure 20:**
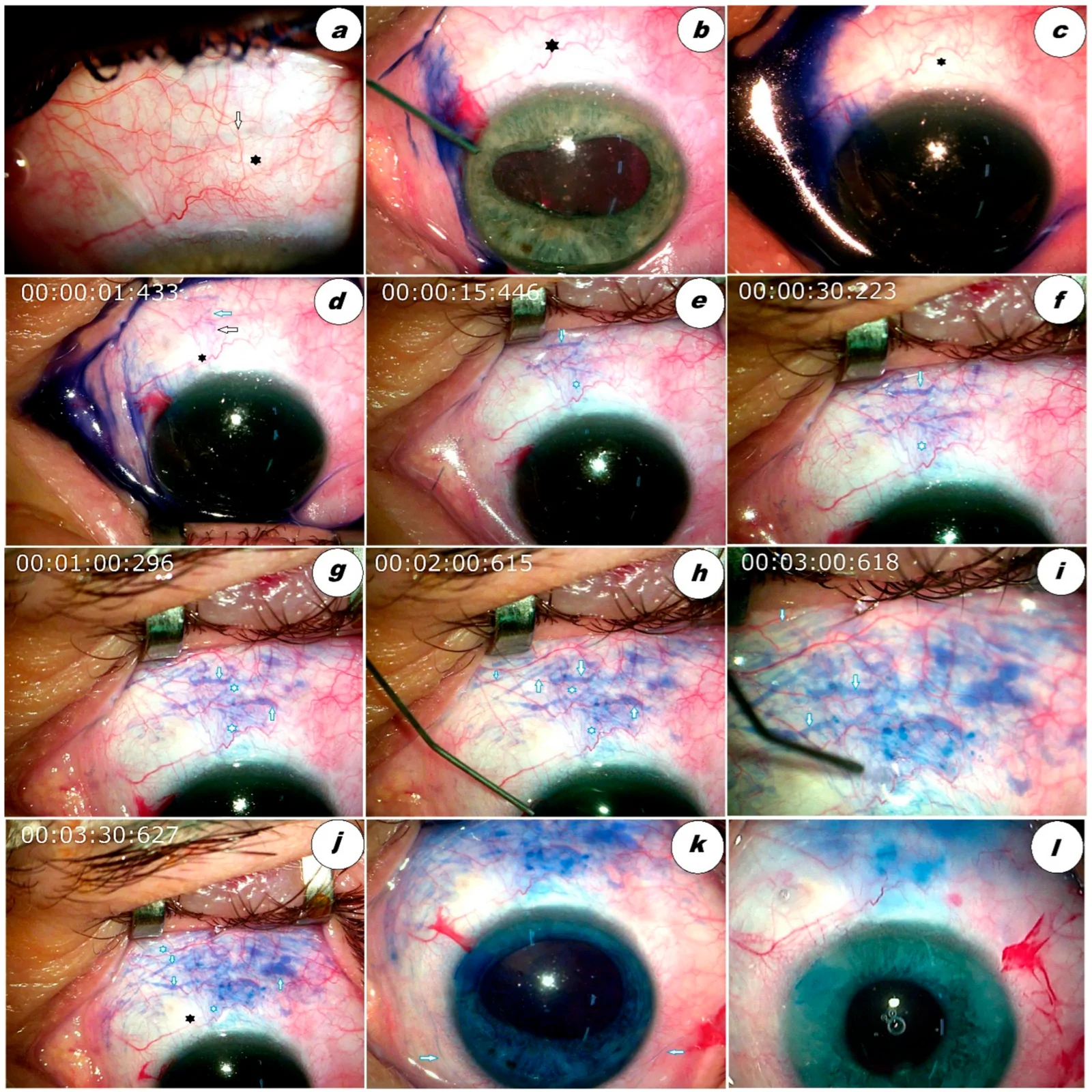
Intraoperative visualization of AH outflow via lymphatic pathways using 0.08% trypan blue as a tracer dye. Note: In all panels, a black star indicates the surgical site; a white arrow with black borders marks the upper border of the superficial scleral flap; a white arrow with turquoise borders indicates lymphatic vessels; a white star with turquoise borders highlights lymphatic plexuses/capillaries. (**a**) Slit-lamp view of the surgical site before cataract surgery in the patient’s left eye, showing absence of blebs at the surgical site (black star). The white arrow with black borders indicates the upper border of the superficial scleral flap. (**b**) Operating microscope view at the start of the study, showing an irregular pupil due to posterior synechiae. A cannula tip is visible in the AC via a paracentesis for AH replacement with trypan blue. (**c**) Complete staining of the AC with 0.08% trypan blue solution. (**d**) Immediately after AC irrigation with the dye, tracer outflow from the AC through an episcleral lymphatic vessel is observed. The vessel emerges 4 mm from the upper limbus (white arrow with black borders: starting point; white arrow with turquoise borders: vessel itself). (**e**–**g**) Progressive appearance and intensification of a lymphatic plexus in the superior bulbar conjunctiva at the temporal side of the surgical site: (**e**) 15 s, (**f**) 30 s, and (**g**) 1 min after AC dye irrigation. CLVs and capillaries become increasingly prominent. (**h**) At 2 min, CLVs occupy a wider area extending toward the medial and temporal sides of the globe. Vessels toward the medial side show sharper contours and brighter color (superficial CLVs), whereas those toward the temporal side have blurred contours and duller color (deeper CLVs). (**i**) Enlarged view at 3 min showing a dense plexus of CLVs and capillaries in the superior bulbar conjunctiva; the surgical field itself remains devoid of these vessels. (**j**) At 3 min 30 s, CLVs of varying calibers and a plexus of capillaries occupy the entire upper segment. Minimal dye leakage into adjacent tissues is observed; virtually all visible dye is confined to the lumens of CLVs. (**k**) At 5 min, the dye has been irrigated out of the AC; CLVs in the superior segment have become less distinct, with mild diffusion of dye into surrounding tissues. Residual dye is visible in thin CLV extending to the inferior fornix (horizontal white arrows with turquoise borders). (**l**) View of the superior conjunctiva at the end of cataract surgery, 20 min after the start of the study. No identifiable CLVs are present; some tissues show residual blue staining.

Case 1. This case illustrates the role of conjunctival lymphatics in suprachoroidal outflow and IOP regulation. A 54-year-old female with glaucoma in the left eye, baseline IOP of 27 mm Hg on three classes of hypotensive medications, and significant intolerance to most IOP-lowering agents underwent surgery in March 2023 using the technique described above. Postoperatively, IOP remained below 13 mm Hg for 10 months without medication or LT. Subsequently, episodic IOP elevation occurred, prompting YAG laser trabeculoplasty. Following LT, IOP normalized and remained within 8–13 mm Hg. Immediately after the procedure, biomicroscopically identifiable CLVs appeared in the superior conjunctiva adjacent to the surgical site; these vessels disappeared within two days. During further follow-up, recurrent IOP spikes were associated with oppositional closure of the trabeculotomy ostium by the iris root. Topical 1% pilocarpine effectively reduced IOP to the single-digit range; however, the patient developed significant intolerance to the medication. To restore a sustained, medication-free aqueous outflow through the trabeculotomy, phacoemulsification with intraocular lens (IOL) implantation was proposed. The rationale was that lens removal and IOL implantation would deepen the AC and eliminate iris-root apposition at the trabeculotomy site. Following detailed discussion, written informed consent was obtained. Prior to phacoemulsification, the AH outflow pathway was assessed intraoperatively. The AC was stained with 0.08% trypan blue as an aqueous tracer dye. The procedure was performed under an operating microscope with video recording capability, and simultaneous video footage of the superior bulbar conjunctiva at and adjacent to the surgical site was obtained. The findings are presented as a series of still frames extracted from the operative video (Figure 20a–l). The video clip is provided as Supplementary Video S8.

**Figure 21:**
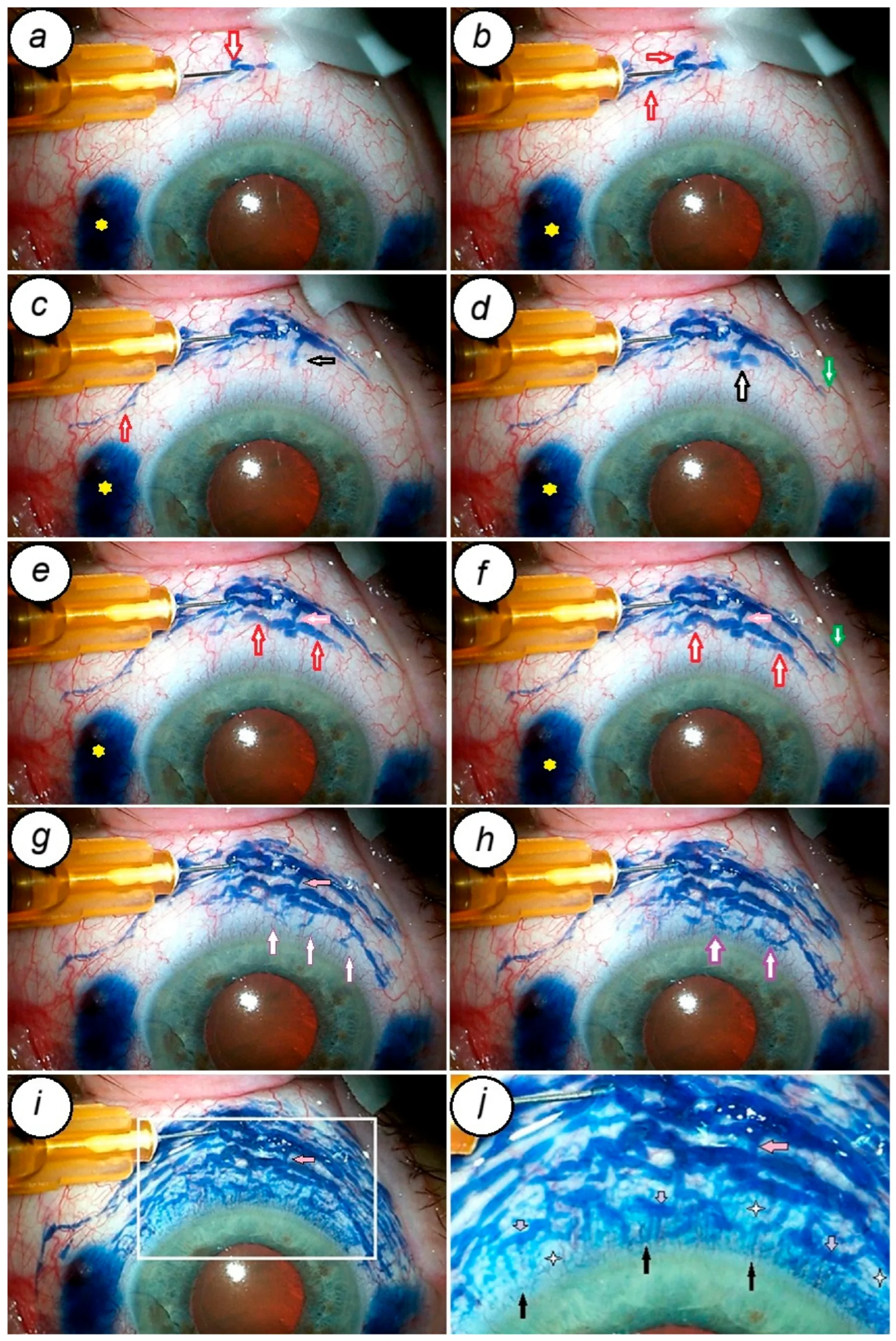
In vivo visualization of the conjunctival lymphatic network during glaucoma surgery. Targeted injection of 0.08% trypan blue into a CLV with a 34G needle resulted in opacification of an extensive lymphatic tree, revealing interconnections between conjunctival, episcleral, and scleral lymphatic pathways. Note: In all panels, the white arrow with red borders indicates the injection site and primary CLV; the pink arrow with black borders marks the connecting “lymphatic bridge”; white arrows with lilac borders indicate limbal lymphatic capillaries; light purple arrows with black borders indicate large limbal capillaries; black arrows indicate small limbal capillaries; white four-pointed stars with black borders indicate episcleral and scleral lymphatic plexuses, the arrow with green border indicates CLV going to fornix, arrow with pink border indicates the connecting lymphatic vessel, yellow star indicates depo from previous investigations, arrow with black border indicates manifestation of CLV of I order. (**a**) Direct injection of trypan blue into a CLV at the 12 o’clock position, 4 mm from the upper limbus. (**b**) Dye filling adjacent CLVs connected to the injected vessel. (**c**,**d**) Progressive filling of the CLV network. The arrow with black borders indicates a vessel that will serve as the sole connecting channel to parallel to limbus CLVs. (**e**,**f**) The connecting “lymphatic bridge” (pink arrow with black borders) linking the superior CLV with the CLV running below, parallel to the limbus. (**g**,**h**) Development of limbal lymphatic capillaries of varying calibers (white arrows with lilac borders) branching from the CLV. (**i**) Overview of the lymphatic tree of the superior bulbar conjunctiva, showing the injected CLV, the lymphatic bridge, multiple layers of the conjunctival lymphatic system, and limbal capillaries. The white rectangle marks the area enlarged in panel j. (**j**) Enlarged view of the area indicated by the white rectangle in (**i**). Small limbal lymphatic capillaries (black arrows) are straight, oriented perpendicular to the corneal edge, with one end connected to large limbal capillaries and the other to episcleral and scleral lymphatic plexuses (white four-pointed stars with black borders). Large limbal lymphatic capillaries (light purple arrows with black borders) run parallel to the limbus, with one blind end and the other connected to the CLV above. The lymphatic bridge is indicated by the pink arrow with black borders. Vessels with poorly defined borders and dull color represent the deep CLV layer, while vessels with sharp contours and bright dye represent superficial CLVs; both layers are interconnected.

Case 2. An anatomical investigation of the CLV was performed using cadaveric specimens, employing an interstitial injection of a modified Gerota mass into the lymphatic and blood vessels to delineate the vascular architecture. This approach, previously applied by Gusev AM, revealed connections between conjunctival and episcleral lymphatic capillaries in the limbal region [11]. In a separate in vivo investigation conducted during glaucoma surgery, the functional lymphatic network was visualized by injecting 0.08% trypan blue into the conjunctival tissue via a 34G needle. The needle directly accessed a CLV; slow dye injection resulted in the opacification of an extensive lymphatic tree, thereby demonstrating the interconnections between different layers of the conjunctival lymphatic system. The observed flow pattern of the tracer and the spatial relationships between CLVs, episcleral, and scleral lymphatics are illustrated in Figure 21a–j. A video recording of the in vivo injection procedure is provided as Supplementary Video S9.

## 5. Discussion

AH circulates within a closed system and is eliminated from the eye predominantly via the conventional outflow pathway. This pathway comprises the trabecular meshwork, SC, collector channels, aqueous veins, and episcleral veins. This outflow pathway is IOP-dependent [1].

Bill et al. were the first to demonstrate the existence of a non-conventional AH outflow pathway. Using radiolabeled tracers of varying molecular sizes, they perfused the AC in multiple animal species (monkeys, rabbits, and cats) to quantify the distribution of tracer outflow [12,13,14,15,16,17,18]. Their findings revealed that approximately 80% of the tracer exited via the trabecular pathway, whereas the remaining fraction accumulated in the ciliary body, choroid, and sclera. Based on these observations, they proposed an alternative route whereby AH flows from the AC through the uvea into the sclera via the choroid and suprachoroid [14,15,17,18]. This pathway is now widely recognized as the uveoscleral outflow route.

Bill and Phillips [19] confirmed the presence of uveoscleral AH outflow in human eyes. Earlier observations were made in animal models (notably in monkeys) [16].

The non-trabecular (uveoscleral/suprachoroidal) outflow pathway involves the ciliary muscle, supraciliary space, and SCS. The supraciliary space is situated anteriorly between the outer surface of the ciliary body and the inner surface of the sclera. The SCS lies posteriorly between the choroid and the inner surface of the sclera [20,21,22].

AH flows from the AC through the most posterior regions of the uveal meshwork, then traverses the interfascial spaces between the longitudinal fibers of the ciliary muscle before entering the supraciliary space and subsequently the SCS.

Morphologically, the SCS is not merely a passive cavity. Karlova et al. [23], who perfused 36 human cadaver eyes with a 5% India ink suspension in balanced saline solution, identified a system of suprachoroidal plates that facilitate unidirectional fluid movement from the posterior edge of the ciliary muscle toward the equatorial region and further to the posterior pole. In their experiments, reverse flow (from the equator toward the limbus) caused the suprachoroidal plates to elevate, effectively preventing anterior fluid migration—a mechanism suggestive of a valvular function. From the ciliary muscle, fluid enters the SCS, where the valvular organization of the suprachoroid promotes unidirectional posterior flow toward the paravascular spaces of the sclera.

Krohn and Bertelsen [24] investigated the morphology of the SCS and potential uveoscleral drainage routes in human donor eyes. India ink-stained gelatin was injected directly into the SCS of seven eyes. Quadrants in which the tracer appeared in the episcleral venous network were serially sectioned and examined by light microscopy. Gelatin was observed in the connective tissue surrounding scleral vessels and nerves, as well as in fine endothelium-lined channels at the inner aspect of the anterior sclera. These channels originated near the scleral spur and communicated with the intrascleral venous plexus. The authors concluded that fluid drains from the SCS through perivascular and perineural spaces of scleral blood vessels and nerves. Their findings also demonstrated the presence of preformed channels at the inner aspect of the anterior sclera capable of draining fluid directly into scleral veins.

In a subsequent study, the same authors confirmed their findings using a corrosion casting technique [25]. Batson’s mixture No. 17 (methyl methacrylate) was injected through a sclerotomy into the SCS of human cadaver eyes. Following polymerization, the surrounding tissue was dissolved with 10% sodium hydroxide, and the casts were examined macroscopically and by scanning electron microscopy. The authors observed transscleral drainage of resin, with several types of branches emerging from the outer (scleral) surface of the casts. Some branches corresponded to perivascular spaces of ciliary vessels and vortex veins. Additionally, branches likely representing channels communicating directly between the SCS and the intrascleral venous plexus were identified.

In a landmark experiment, Bill [26] ligated the major cervical lymphatic vessels and all connections between cervical and thoracic lymphatics near the thoracic inlet of a rabbit. After ligation, the cervical lymphatics on the cranial side of the ligatures were opened to allow free lymph outflow. A 2 μL volume of labeled albumin solution was then injected into the SCS. The author concluded that albumin left the SCS by bulk flow, with more than 85% passing transsclerally and being collected by lymphatic vessels. Bill also noted that diverting the cervical lymphatic pathways from the major veins did not completely prevent labeled albumin from entering the systemic circulation, as approximately 15% still reached the bloodstream.

The non-trabecular (uveoscleral) outflow pathway is regulated by the resistance imposed by the ciliary muscle bundles and the connective tissue of the ciliary body [27]. Notably, flow through this route is largely pressure-independent under physiological conditions. A modest pressure gradient exists between the AC and the supraciliary/SCS, with pressures in these spaces being slightly lower than IOP. Consequently, an increase in AC pressure has minimal effect on the overall pressure gradient and does not substantially augment uveoscleral outflow.

In contrast, surgical disruption of the normal anatomical barrier—such as in cyclodialysis—creates a pressure-dependent bypass that circumvents the resistance normally imposed by the ciliary muscle bundles. By converting the uveoscleral outflow route into a pressure-dependent pathway, this intervention markedly augments AH drainage, resulting in pronounced IOP reduction. Postoperatively, IOP levels may decline to the low teens (10–13 mm Hg) or even to single digits (<10 mm Hg), underscoring the substantial impact of altering outflow dynamics [28].

AH exits the SCS via the following three identified routes: uveoscleral, uveovortex, and uveolymphatic [13,29,30]. It has been established that uveoscleral outflow is critically dependent on scleral permeability [1]. This property influences the transscleral movement of AH and, consequently, the efficiency of non-trabecular drainage. A fraction of the fluid within the SCS is proposed to be absorbed osmotically by the choroid and subsequently drain into the vortex veins via the uveovortex pathway [29,31,32]. This outflow mechanism is driven by a substantial colloidal osmotic gradient. The contributions of Barany [29], Pederson et al. [31], and Sherman et al. [32] collectively support the existence of this route, highlighting the choroid not only as a vascular bed but also as an active absorptive interface in AH dynamics.

Yucel et al. [30,33] provided histological evidence for a uveolymphatic pathway for AH drainage by demonstrating lymphatic endothelium in the human ciliary body using specific lymphatic markers, including podoplanin (a transmembrane mucin-type glycoprotein) and lymphatic vessel endothelial hyaluronan receptor-1 (LYVE-1). These findings were subsequently corroborated by Oatts and colleagues [34], who compared the uveoscleral outflow capability of two different suprachoroidal shunts under both in vitro and in vivo conditions, thereby supporting the functional relevance of the uveolymphatic route.

The supraciliary space has emerged as a therapeutically relevant target for IOP reduction in glaucoma management. Various surgical techniques have been developed to enhance AH flow through the non-trabecular outflow pathway by accessing this space [35]. A key advantage of these approaches is their blebless nature, which addresses several limitations associated with traditional filtering surgeries.

In contrast to conventional procedures, which rely on subconjunctival bleb formation, blebless supraciliary interventions are associated with a reduced risk of flat AC, minimized tissue trauma, and lower rates of postoperative complications. Notably, the absence of a filtering bleb eliminates risks such as bleb leakage and lifelong susceptibility to endophthalmitis, while also mitigating the unpredictability of wound healing responses [35]. Furthermore, by rerouting AH directly from the AC to the supraciliary or SCS, these procedures significantly reduce inflammation and fibrosis, thereby decreasing the likelihood of surgical failure [34].

An additional clinical benefit is the flexibility in surgical approach: the supraciliary space can be accessed via either an ab externo or an ab interno route, allowing surgeons to tailor the technique to individual patient factors.

Various surgical devices have been developed to exploit the uveoscleral outflow pathway for IOP reduction in glaucoma, including the SOLX Gold Shunt™ [36,37,38,39], the polypropylene Aquashunt™ [34], and the STARflo™ [40]. These blebless implants are designed to enhance non-trabecular outflow without inducing cyclodialysis. Classified as penetrating glaucoma procedures, they are positioned so that one end communicates with the AC and the other with the SCS, thereby creating a controlled fluid conduit between the two compartments. Several studies have demonstrated that fibroblastic activation represents a principal cause of surgical failure for supraciliary shunt procedures [34,41,42,43].

Prior studies have sought to enhance the outcomes of deep sclerectomy (DS) by augmenting supraciliary outflow [36,44,45,46,47,48,49,50,51]. Although these efforts have yielded encouraging results, the efficacy of such modifications remains poorly defined.

Deep sclerectomy is a non-penetrating filtering procedure in which AH drains to the subconjunctival space, resulting in bleb formation. The reliance on a conjunctival bleb as the primary filtration site may limit the full exploitation of the non-trabecular outflow pathway, potentially compromising final outcomes. This consideration underscores the need to prevent AH leakage into the subconjunctival space to maximize utilization of alternative drainage routes.

The technique described herein addresses both objectives. Watertight suturing of the superficial scleral flap, combined with a sufficient interval for complete healing of the conjunctiva to the underlying tissues, effectively prevents subconjunctival AH leakage. A collagen implant placed in the intrascleral channel (ISC) and the suprachoroidal tunnel serves dual functions: as a spacer to maintain space and as a conduit to facilitate AH flow. Once the conjunctiva is fully healed, the ISC is separated from the AC only by the thin inner wall of SC. Following LT, AH flow is rerouted exclusively from the AC to the SCS.

Several recent studies have reported encouraging outcomes of deep sclerectomy (DS) augmented with suprachoroidal drainage to improve long-term efficacy [52,53,54,55]. Various biomaterials have been proposed to maintain the cyclodialysis cleft, including autologous sclera [6,7], collagen-based implants (e.g., Ologen^®^) [4,56], and synthetic materials such as Teflon [57,58,59], polyethylene, silicone, silicone elastomer (Silastic^®^) [5], hydrogel [60], and metallic devices [3,61]. These implants can be placed via either an ab externo or an ab interno approach. However, clinical outcomes with these devices have been generally disappointing, offering no demonstrable advantage over established techniques.

The contribution of lymphatic drainage to AH outflow has been documented in both animal models and healthy human subjects following penetrating, non-penetrating, and minimally invasive glaucoma surgeries (MIGSs) [62,63,64,65,66,67,68,69]. Recent evidence further demonstrates that the lymphatic system plays a significant role in trabecular outflow, in addition to the blood circulation system [70].

Historically, the conventional outflow pathway was considered to terminate from SC directly into the episcleral veins, with no recognized connection to lymphatic vessels. This paradigm has been challenged by Yang et al. [70], who identified a novel anatomical structure—termed the “lymphatic bridge”—that directly links the SC to the limbal and conjunctival lymphatic pathways. Using high-resolution three-dimensional (3D) imaging of intact globes, the authors demonstrated the existence of a new conjunctival outflow route for AH from the AC.

Under normal conditions, the non-trabecular outflow accounts for less than 20% of the total AH outflow. This proportion increases substantially when the resistance of the ciliary muscle is bypassed, resulting in a marked augmentation of uveoscleral outflow. The mechanisms responsible for handling the increased fluid load within the SCS remain unclear. Conventional drainage pathways are insufficient to accommodate the excess fluid, suggesting the potential involvement of additional compensatory mechanisms for its clearance.

Except for a few reports on the role of lymphatics in suprachoroidal drainage to maintain post-surgical hydrodynamics [8,9,30], this question has not received proper attention. Sauntharrajan B et al. [71] used near-infrared (NIR) fluorescence imaging to detect the NIR fluorescent tracer CF770/BSA 20 min after suprachoroidal injection in adult mice. Suprachoroidally injected tracer was detected within podoplanin-positive CLVs in the nasal region of the ipsilateral eye, with minimal signal observed in contralateral control eyes. The authors concluded that CLVs participate in distal drainage of tracer from the SCS.

Khoo et al. [69], using trypan blue as an aqueous tracer to diagnose cyclodialysis clefts in persistent hypotony, incidentally identified “lymphatic overdrain” as a cause of post-trabeculectomy hypotony. Of 10 hypotonous subjects, seven had confirmed cyclodialysis clefts with preferential dye flow to the cleft region. The remaining three included two post-trabeculectomy patients who exhibited rapid (within 5 s) and extensive lymphatic staining spanning over six clock hours without visible bleb formation—a finding the authors termed “lymphatic overdrain.” Both patients were female with an IOP of 1 mmHg, hypotonous maculopathy, and no visible trabeculectomy bleb. After the dye study, both underwent targeted intervention: one underwent conjunctival dissection and diathermy at the site of lymphatic overdrain, and the other received direct diathermy to the conjunctiva at the lymphatic origin from the sclera. In both patients, IOP normalized and visual acuity improved. The authors proposed that a direct lymphatic connection to AH had been established beneath the trabeculectomy scleral flap, removing AH faster than it could accumulate in the subconjunctival space, thereby preventing bleb formation. Diathermy disrupted this lymphatic–intrascleral connection in both cases.

In this report, clinical observations supported by OCT imaging described the role of scleral, episcleral, and CLVs in suprachoroidal drainage. A notable finding was that CLVs arose directly from the sclera at sites distant from the surgical site, with no connection to the surgical site. This suggests that AH did not preferentially accumulate in the SCS at the surgical site but spread to other regions. Upon reaching scleral areas rich in lymphatic vessels, AH exited the SCS, entered the scleral lymphatics, and continued through episcleral lymphatic vessels to reach the CLVs—consistent with the proposed SCS–scleral–episcleral–CLV outflow route. An alternative explanation is that intraoperative diathermy, applied to the superficial scleral layers for hemostasis, may have damaged CLVs at the surgical site, redirecting AH drainage through alternative scleral exit pathways.

In the present case series, clinical observations and OCT evaluation of the surgical sites confirmed the absence of FB in all cases, indicating that the proposed technique enables most of the AH to flow from the AC to the SCS without being diverted to the subconjunctival space. The results support the role of lymphatics in suprachoroidal drainage and IOP reduction. In more than 50% of cases, previously nonvisible CLVs became visible biomicroscopically, indicating their involvement in AH drainage. The time of their appearance, along with their duration, number, shape, and size, varied from patient to patient. In certain cases, they appeared days to weeks after surgery, while in others, they appeared immediately after LT—especially in cases with higher postoperative IOP. Observations indicate that to activate this pathway, LT should be performed when the patient’s IOP is above the target IOP. These findings support the observations of Benedikt [64], who noted that if the IOP is too low at the surgery site after filtering surgery, in most cases a FB will form. If the IOP is above the episcleral vein pressure, new vessels, including blood and lymphatic vessels, may develop and drain the AH by bulk flow from the scleral fistula, providing a good pressure-regulating effect without a filtering bleb.

A reason for the biomicroscopic appearance of CLVs in some cases, especially in patients who underwent LT, was proposed in our previous report [9]. The results of this study further support that hypothesis. In living organisms, the lymphatic vasculature plays an important role in regulating interstitial fluid homeostasis by draining excess interstitial fluid. Extracellular fluid, leukocytes, proteins, and metabolites enter blind-ended, permeable lymphatic capillaries to form lymph. The conjunctiva, episclera, and sclera contain numerous preexisting, quiescent lymphatic vessels. Following surgery using the proposed technique and after LT, AH enters the ISR and accumulates at a pressure exceeding the episcleral venous pressure. From there, AH enters the SCS, facilitated by a BCI positioned with one end in the ISR and the other end in the suprachoroidal tunnel. The BCI serves not only as a spacer but also as a conduit. Because there is no leakage into the subconjunctival space, AH enters the SCS exclusively. From there, through scleral microchannels and lymphatics, AH exits the SCS and enters the episcleral and CLVs. With an excessive volume of AH in their lumens, the CLVs and capillaries become engorged. They remain in this state if forward lymph flow is inadequate or resistance is encountered along the drainage pathway. The engorgement of vessels with excess AH renders them visible on slit-lamp examination. With the establishment of normal AH flow through these vessels, the CLVs become less prominent and decrease in size and number. At this stage, they are no longer visible on biomicroscopy but remain identifiable on OCT. This was demonstrated in our previous reports and is further supported by the present findings. These results demonstrate that continuous AH flow from the AC to the SCS activates a novel outflow pathway—the SCS–scleral–episcleral–CLV outflow route—to drain excess fluid from the SCS.

In this series, BCI was used as a spacer and conduit. Several authors have used intrascleral implantation of collagen implants to enhance the success rate of DS and to reduce fibrosis at the surgical site [46,72,73]. These implants maintain the space in the scleral bed, provide support for the AH drainage route, and act as a sponge, transporting fluid by capillary action. Mitwally et al. [50] demonstrated in a randomized controlled trial that suprachoroidal CI implantation combined with DS produced a greater IOP-lowering effect than intrascleral implantation. Shradqa et al. [4] studied the role of a non-absorbable collagen implant (Xenoplast, Dubna-Biofarm, Moscow, Russia) in the SCS for IOP reduction. According to these authors, Xenoplast may be implanted via an ab externo or ab interno approach. The main drawback of this implant is its coarse surface, which traumatizes uveal tissue during implantation, leading to fibrosis and blockage of the cyclodialysis tunnel. The implant used in the present study is thin and stiff in its dry state, with smooth surfaces, making implantation easy and atraumatic. Notably, in the proposed technique, exposure of the uveal tissue surface for AH resorption is maximized by incising an additional strip of the deep scleral layers posterior to the scleral bridge. The specific BCI placement used in this technique plays an important role in activating the uveolymphatic outflow pathway.

The proposed technique has certain advantages. All surgical steps are performed without perforating the eyeball; hence, complications associated with a sudden decrease in IOP are minimized or avoided. In this method, the enhanced AH outflow occurs through the SCS–scleral–episcleral–CLV route, functioning as a bleb-independent drainage pathway. This outflow is independent of FB. Consequently, there is no need for antifibrotic agents to prevent bleb fibrosis and enhance bleb survival, nor for needling or revision of failed blebs. Furthermore, bleb-related complications such as bleb leaks, infection, and dysesthesia—well-recognized sources of morbidity after conventional filtering surgery—are inherently avoided.

This technique has certain drawbacks as well. First, it is an ab externo procedure that requires extensive tissue dissection, resulting in surgical trauma and increased operative time. The technique also involves a steep learning curve, as precise deep scleral dissection and suprachoroidal tunnel creation demand significant surgical experience. Second, it is a two-stage surgery; the interval between stages necessitates close monitoring of patients in the early postoperative period, as IOP may remain elevated until LT is performed. Third, there is a possibility that the trabeculotomy opening will be blocked by iris tissue, resulting in an increase in IOP. Performing a peripheral iridotomy before, during, or after surgery can help prevent this complication. Fourth, although the technique avoids bleb-related fibrosis, fibrotic encapsulation around the BCI or within the suprachoroidal tunnel remains a potential cause of late failure, as has been reported with other suprachoroidal implants. Fifth, the technique depends on functional scleral, episcleral, and conjunctival lymphatic pathways; patients with compromised lymphatics due to prior conjunctival surgery, trauma, or chronic inflammation may not respond adequately. Finally, the findings are based on a limited case series with a relatively short follow-up period, and the long-term durability of the lymphatic outflow pathway remains to be established.

This study has several limitations. It is a single-center consecutive case series without a control group, involving a relatively small cohort and a limited follow-up period, which constrains the evaluation of long-term outcomes and rare adverse events. The progressive loss to follow-up across visits may have introduced selection bias and affected the power of the analysis. Additionally, the assessment of CLVs was based on biomicroscopic visibility, which is subjective and not supported by standardized grading criteria. IOP was measured at single time points without diurnal variation curves, potentially affecting the accuracy of success classification, particularly in eyes with borderline IOP. Furthermore, outcome assessment was performed by the operating surgeon, introducing potential observer bias. The results may also be influenced by surgical expertise at a single center and may not be generalizable to other settings. Subgroup analyses (e.g., by prior glaucoma surgery, baseline IOP, or severity of disease) were not performed, limiting the ability to identify predictors of response. Future randomized controlled trials with larger, multicenter cohorts, masked outcome assessment, and standardized imaging protocols are needed to further evaluate the efficacy, safety, and reproducibility of this technique.

## 6. Conclusions

The long-term clinical outcomes of this study, with follow-up extending to 36 months, demonstrate that the proposed technique is safe and effective in reducing IOP and reducing the medication burden, without FB formation in any case. A possible novel outflow route—the SCS–scleral–episcleral–CLV pathway—was identified for suprachoroidal drainage, supported by both clinical observation and OCT findings. By eliminating dependence on FB, this approach circumvents bleb-related complications and the need for antifibrotic agents, representing a promising alternative in the surgical management of glaucoma.

## 7. Patents

1. Method to activate the uveoscleral outflow pathway of aqueous humor. Patent of the Russian Federation for invention No. 2712640, granted on 30 January 2020.
2. Combined method to activate different mechanisms of aqueous humor outflow in glaucoma. Patent of the Russian Federation for invention No. 2766730, granted on 15 March 2022.
3. Surgical method for simultaneous activation of different aqueous humor outflow pathways in glaucoma. Patent of the Russian Federation for invention No. 2782126 granted on 21 October 2022.
4. Method to treat open angle glaucoma. Patent of the Russian Federation for invention No. 2830376, granted on 18 November 2024.
5. Method to activate the conventional and non-conventional outflow pathways of aqueous humor in glaucoma. Patent of the Russian Federation for invention No. 2833766, granted on 28 January 2025.

## Figures and Tables

**Table 1:** Demographic and baseline clinical characteristics of the study population.

Variable n (%)	Overall (n = 70)	Standalone Surgery Group (n = 22)	Combined Surgery Group (n = 48)	*p*-Value	Test
Age, years	75.0 ± 7.3	76.9 ± 6.2	74.2 ± 7.6	0.117	Welch’s *t*-test
Sex, n (%)					Fisher’s exact
Male	21 (30.0)	7 (31.8)	14 (29.2)	1.000	
Female	49 (70.0)	15 (68.2)	34 (70.8)	1.000	
Eye, n (%)					Fisher’s exact
Right (OD)	33 (47.1)	10 (45.5)	23 (47.9)	1.000	
Left (OS)	37 (52.9)	12 (54.5)	25 (52.1)	1.000	
Follow-up, months median, (IQR)	17.5 [14.1; 36.6]	25.8 [11.6; 35.5]	16.4 [14.2; 36.8]	0.761	Mann–Whitney U
Glaucoma type, n (%)					Fisher’s exact
Non-refractory open-angle glaucoma	53 (75.7)	10 (45.5)	43 (89.6)	<0.001	
Refractory open angle glaucoma	17 (24.3)	12 (54.5)	5 (10.4)	<0.001	
Severity, n (%)					Fisher’s exact
Moderate	21 (30.0)	6 (27.3)	15 (31.2)	0.787	
Severe	49 (70.0)	16 (72.7)	33 (68.8)	0.787	
Prior glaucoma surgery, n (%)					Fisher’s exact
Yes	18 (25.7)	11 (50.0)	7 (14.6)	0.003	
No	52 (74.3)	11 (50.0)	41 (85.4)	0.003	
Trabeculectomy, n	7	4	3		
Deep sclerectomy, n	2	2	0		
Laser iridotomy, n	2	2	0		
SC stenting, n	2	0	2		
Modified deep sclerectomy with “Xenoplast” as space maintainer, n	5	3	2		
Preoperative IOP mmHg, mean ± SD	28.8 ± 8.4	31.3 ± 10.1	27.7 ± 7.4	*p* = 0.092, *p* = 0.068)	*t*-test; Mann–Whitney
Preoperative medication, mean ± SD; median (IQR)	2.77 ± 0.94; 3.0 (2.0–3.0).	3.05 ± 0.79; 3.0, (2.25–4.0)	2.65 ± 0.98; 3.0, (2.0–3.0)	(*p* = 0.097; *p* = 0.128)	*t*-test; Mann–Whitney
1 medication, n (%)	7 eyes (10%),				
2 medications, n (%)	19 eyes (27.1%),				
3 medications, n (%)	27 (38.6%),				
4 medications, n (%)	17 (24.3%).				
Pseudophakia, n (%)					Fisher’s exact
Yes	21 (30.0)	21 (95.5)	0 (0.0)	<0.001	
No	49 (70.0)	1 (4.5)	48 (100.0)	<0.001	
Comorbidities, n					
Wet AMD	1	1			
Dry AMD-1 eye, epiretinal membrane -1 eye	1	1			
Mild myopia	0	1			
High myopia	0	4			
Diabetes mellitus	2	3			

AMD = age-related macular degeneration; IOP = intraocular pressure; n = number of eyes; IQR = interquartile range, SD = standard deviation. Fixed-dose combinations were counted as one medication but attributed to each component class for class-level reporting.

**Table 2:** IOP mixed-effects model (adjusted for baseline IOP).

Model	Parameter	Estimate (95% CI)	*p*-Value	Comments
Whole cohort	Baseline IOP	0.188 (0.098–0.278)	<0.001	Higher starting IOP → higher follow-up IOP
	Time (per month)	−0.068 (−0.125 to −0.011)	0.020	IOP drops ~0.8 mmHg/year
Standalone surgery group	Baseline IOP	0.099 (−0.038 to 0.236)	0.159	Not a significant predictor
	Time (per month)	−0.109 (−0.195 to −0.023)	0.015	IOP drops ~1.3 mmHg/year
Combined surgery group	Baseline IOP	0.257 (0.129–0.385)	<0.001	Each +1 mmHg baseline → +0.26 mmHg follow-up
	Time (per month)	−0.042 (−0.121 to 0.036)	0.301	IOP stable after surgery
Interaction	Group (2 vs. 1)	−0.945 (−2.793 to 0.903)	0.317	No difference in IOP level
	Time × group	0.076 (−0.043 to 0.195)	0.214	No difference in IOP slope

**Table 3:** Visual acuity (logMAR)—mixed-effects model (adjusted for baseline VA).

Model	Parameter	Estimate (95% CI)	*p*-Value	Comments
Whole cohort	Baseline VA	0.762 (0.612–0.912)	<0.001	Higher baseline VA → higher follow-up VA
	Time (per month)	0.000 (−0.002 to 0.002)	0.848	No significant change over time
Standalone surgery group	Baseline VA	0.845 (0.651–1.039)	<0.001	Significant covariate
	Time (per month)	0.001 (−0.003 to 0.005)	0.787	Stable VA
Combined surgery group	Baseline VA	0.724 (0.555–0.893)	<0.001	Significant covariate
	Time (per month)	−0.001 (−0.003 to 0.002)	0.831	Stable VA
Interaction	Group (2 vs. 1)	−0.121 (−0.345 to 0.103)	0.294	No difference in VA level
	Time × group	−0.002 (−0.006 to 0.003)	0.442	No difference in VA slope

Random intercept per eye; REML estimation. Models adjusted for baseline VA.

**Table 4:** Distribution of visible CLVs in the superior bulbar conjunctiva, as observed on slit-lamp biomicroscopy.

	CLV Visualization On Slit-Lamp Biomicroscopy					
Site of CLV visualization	SS	SN	ST	SS + SN	SS + ST	SN + ST
Number of observations	5	46	17	6	6	7

SS = surgical site; SN = superior nasal quadrant; ST = superior temporal quadrant. Values are expressed as number of observations (not number of eyes). Multiple observations were possible per eye across different follow-up visits.

## Data Availability

The original contributions presented in this study are included in the article/Supplementary Materials. Further inquiries can be directed to the corresponding author.

## Supplementary Materials

The following supporting information can be downloaded at https://www.mdpi.com/article/10.3390/vision10040084/s1, Video S1: Peripheral iridotomy using capsulorhexis needle with Oertli CataRhex 3 apparatus. Video S2: Surgical technique. Video S3: Insertion of two small pieces of 4-0 polypropylene suture in the lumen of Schlemm’s canal. Video S4: Biomicroscopic visualization of CLVs by moving eye lid margin over conjunctiva applying resistance to lymph flow. Video S5: Biomicroscopic visualization of CLV immediately after LT. Video S6: Biomicroscopic visualization of different patterns of CLV. Video S7: Biomicroscopic visualization of deep CLV running parallel to limbus. Video S8: Demonstrating the role of lymphatics in suprachoroidal drainage using a trypan blue aqueous tracer dye to investigate the possible AH outflow route after surgery. Video S9: In vivo investigation of conjunctival lymphatic vessels at the time of glaucoma surgery, with direct puncture of the vessel and manifestation of the lymphatic tree.
